# Prevalence of schizophrenia in China between 1990 and 2010

**DOI:** 10.7189/jogh.05.010410

**Published:** 2015-06

**Authors:** Kit Yee Chan, Fei–fei Zhao, Shijiao Meng, Alessandro R Demaio, Craig Reed, Evropi Theodoratou, Harry Campbell, Wei Wang, Igor Rudan, Global Health Epidemiology Reference Group (GHERG)

**Affiliations:** * Joint first authors; ** Joint senior authors; 1 Centre for Population Health Sciences and Global Health Academy, University of Edinburgh Medical School, Scotland, UK; 2 World Health Organization’s Collaborative Centre for Population Health Research and Training; 3 Nossal Institute for Global Health, University of Melbourne, Australia; 4 Municipal Key Laboratory of Clinical Epidemiology, School of Public Health, Capital Medical University, Beijing, China; 5 Harvard Global Equity Initiative, Harvard Medical School, Boston, USA; 6 Copenhagen School of Global Health, University of Copenhagen, Denmark; 7 School of Medical Sciences, Edith Cowan University, Perth, Australia

## Abstract

**Background:**

Dramatic development and changes in lifestyle in many low and middle–income countries (LMIC) over the past three decades may have affected mental health of their populations. Being the largest country and having the most striking record of development, industrialization and urbanization, China provides an important opportunity for studying the nature and magnitude of possible effects.

**Methods:**

We reviewed CNKI, WanFang and PubMed databases for epidemiological studies of schizophrenia in mainland China published between 1990 and 2010. We identified 42 studies that reported schizophrenia prevalence using internationally recognized diagnostic criteria, with breakdown by rural and urban residency. The analysis involved a total of 2 284 957 persons, with 10 506 diagnosed with schizophrenia. Bayesian methods were used to estimate the probability of case of schizophrenia (“prevalence”) by type of residency in different years.

**Findings:**

In urban China, lifetime prevalence was 0.39% (0.37–0.41%) in 1990, 0.57% (0.55–0.59%) in 2000 and 0.83% (0.75–0.91%) in 2010. In rural areas, the corresponding rates were 0.37% (0.34–0.40%), 0.43% (0.42–0.44%) and 0.50% (0.47–0.53%). In 1990 there were 3.09 (2.87–3.32) million people in China affected with schizophrenia during their lifetime. The number of cases rose to 7.16 (6.57–7.75) million in 2010, a 132% increase, while the total population increased by 18%. The contribution of cases from urban areas to the overall burden increased from 27% in 1990 to 62% in 2010.

**Conclusions:**

The prevalence of schizophrenia in China has more than doubled between 1990 and 2010, with rates being particularly high in the most developed areas of modern China. This has broad implications, as the ongoing development in LMIC countries may be increasing the global prevalence of schizophrenia.

Schizophrenia is a complex psychiatric disorder with devastating costs to both patients and societies [1,2]. The etiology of schizophrenia is still not understood, but both genetic, environmental and behavioral risk factors have been proposed, including development, industrialization and urbanization [3,4]. Dramatic development and changes in lifestyle in many low and middle–income countries (LMIC) over the past three decades may have affected mental health of their populations. Being the largest country and having the most striking record of development and urbanization, China provides an important opportunity for studying the nature and magnitude of possible effects [4–12]. The mechanisms through which development and urbanization increase schizophrenia risk are not understood [5–7]. Several hypotheses have been proposed, but presently there is insufficient evidence in their support [6–9]. Exploring them further remains a significant challenge, which is particularly true in LMIC where the availability and quality of epidemiological information is often suboptimal [13].

Epidemiological and demographic evidence in China has improved over the past two decades and Chinese academic journals have become accessible in electronic databases such as China National Knowledge Infrastructure (CNKI) and Wanfang [14–18]. Demographic data imply that the proportion of Chinese population living in urban areas has increased from about one quarter to one half between 1990 and 2010 [18]. We may therefore expect a significant increase in both the prevalence and the absolute number of cases of schizophrenia in China over the past two decades, along with a growing population–attributable fraction assigned to development, industrialization and urbanicity. To explore this, we conducted a systematic review of the literature in Chinese and English to analyze trends in the prevalence of schizophrenia in China over the 20–year period from 1990 to 2010 in urban and rural regions. Any changes observed in China may also have relevance for many other LMIC with large rural–to–urban migration associated with industrialization.

## METHODS

### Literature search strategy and search terms

Systematic reviews of China National Knowledge Infrastructure (CNKI), Wanfang and PubMed were conducted for the publication years 1990 to 2010. Searches of the Chinese databases were performed independently by two co–authors (FFZ and SJM), and subsequent PubMed searches by two further co–authors (ARD and KYC). Table s1 in **Online Supplementary Document(Online Supplementary Document)(Online Supplementary Document)** shows the search terms for the Chinese databases, which consisted of the Chinese and English terms for schizophrenia searched in combination with each of the following: epi* (all Chinese terms for epidemiology), incidence (in two Chinese variants), cross–sectional study (in two Chinese variants), prevalence, point prevalence, mortality, case–fatality and attack rate. Based on the results of the detailed Chinese searches, the search terms for PubMed were reduced to “schizophrenia AND China AND (inciden* OR prevalence OR morbidity OR mortality)”.

### Inclusion and exclusion criteria

**Figure 1:**
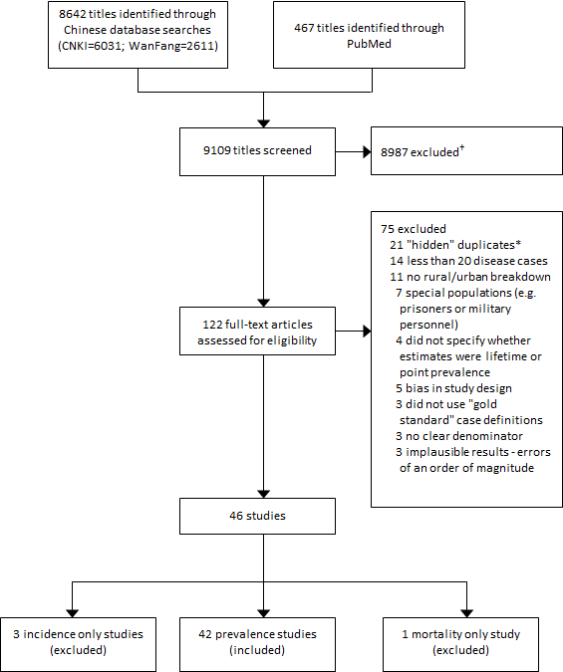
PRISMA diagram of the study selection. †The large majority was excluded because they were duplicate returns of the same reference under different search terms or in different databases; the others were excluded because they were irrelevant to the topic of our study, provided no numerical estimates, or studied Chinese populations outside of mainland China. *Reports of the same results in different journals.

The two Chinese databases initially yielded a total of 8642 titles, while PubMed yielded 467. Papers were first excluded on the basis of duplicate publications within and between databases, studies with no numerical estimates, studies of Chinese population outside of mainland China, reviews, and viewpoints, reducing the yield to 122 full–text papers. Further studies were excluded if they reported less than 20 schizophrenia cases, had no clear denominator or were not representative of the general population. In reviewing the methods of each paper further exclusions were made on the basis that the paper did not provide a clear differentiation between rural and urban residents or had failed to specify whether the reported prevalence was lifetime or point prevalence. Finally, after checking the case definition used in each paper, only papers that had applied a ‘gold standard’ case definition were retained (ie, Diagnostic and Statistical Manual of Mental Disorders (DSM)–III or IV, International Classification of Diseases (ICD)–9 or 10, or Chinese Classification of Mental Disorders – CCMD–II and above). Four papers were excluded because they reported only incidence or mortality rates with no prevalence estimates. Direct contact was made with the corresponding authors of 13 of the retained studies to obtain missing information related to the inclusion criteria, thus removing any ambiguities about the studies. Publications from the same field site that reported partial results were merged and counted as one study. **Figure 1** shows the PRISMA (acronym for: preferred reporting items for systematic reviews and meta–analyses) diagram illustrating the process of selection of studies.

### Geographic location and study year of the retained studies

**Figure 2:**
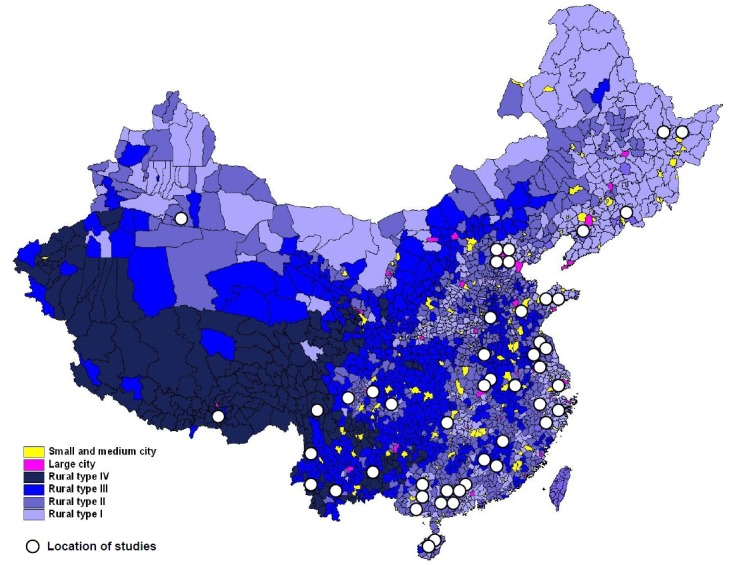
Geographic location of 48 study sites in 42 retained studies. The studies are shown on the map of China that represents the areas of urban and rural areas (I = most developed; IV = least developed) around the mid–point of the study period.

After all exclusions, 42 prevalence studies were retained. Their full references are presented in Table s2 in **Online Supplementary Document(Online Supplementary Document)**. The key characteristics of the studies are summarized in **Table 1**. The studies were mostly large population–based studies. They typically used a two–stage data collection design in which trained medical assessors performed an initial population–based screening in Phase 1 and psychiatrists performed a detailed evaluation in Phase 2 (see Table s3 in **Online Supplementary Document(Online Supplementary Document)** for further explanation on study design, agreement statistics and validation of the estimates). We defined “year of study” as the median year of the exact period during which the study was conducted. Geographically, the retained studies covered 21 of mainland China’s 31 provinces, municipalities and autonomous regions (Table s4 in **Online Supplementary Document(Online Supplementary Document)**). Their geographic distribution is shown in **Figure 2****.**

**Table 1:** Characteristics of the cross–sectional studies

Study characteristics	Number of studies (n = 42)
**Sample size (n):**	
<5000	8 (19.0%)
5001–10 000	9 (21.4%)
10 001–20 000	11 (26.2%)
20 001–60 000	7 (16.7%)
>60 000	7 (16.7%)
**Year published:**	
1990–1994	6 (14.3%)
1995–1999	6 (14.3%)
2000–2004	15 (35.7%)
2005–2010	13 (31.0%)
2011	2 (4.8%)
**Setting:**	
Urban	4 (9.5%)
Rural	10 (23.8%)
Both	28 (66.7%)
Case Definitions*	
Chinese Classification of Mental Disorders (CCMD)–II:	9 (21.4%)
CCMD–II–R	11 (26.2%)
CCMD–III	8 (19.1%)
DSM–III–R	4 (9.5%)
DSM–IV	4 (9.5%)
ICD–9	2 (4.8%)
ICD–10	10 (23.8%)

DSM – Diagnostic and Statistical Manual of Mental Disorders, ICD – International Classification of Diseases

*Proportions do not add up to 100% because several studies used both CCMD and DSM/ICD case definition.

### Study design

The main aim of this study was to estimate the prevalence of schizophrenia in China over two decades of intense development (1990–2010). China is a particularly appropriate setting for studying the effect of development, industrialization and urbanization because of a well–documented administrative categorization of its population into rural and urban areas, thus preventing major misclassification [19–21]. We therefore considered estimates of prevalence (point and lifetime) to be the dependent variable, while the year of study and urban/rural residency were the key predictors. We relied on point prevalence as the primary outcome for testing the hypothesis on the effect of development on the prevalence of schizophrenia. This is because point prevalence was based on direct assessment of study subjects, while lifetime prevalence was prone to self–assessment bias and recall bias. Therefore, we considered point prevalence a more reliable indicator of disease frequency for the purpose of hypothesis testing. However, we used lifetime prevalence in assessing the population–attributable risk (PAR) associated with development, industrialization and urbanization, because it was consistently larger than point prevalence and it therefore provided more useful policy–relevant information. Relying on point prevalence for this purpose would lead to an underestimate of the size of the problem in China.

### Statistical analyses

Based on the retained 42 studies, Bayesian methods (see eMethods in **Online Supplementary Document(Online Supplementary Document)**) were applied to predict maximum likelihood for point prevalence and lifetime prevalence in urban and rural areas of China in the years 1990, 2000 and 2010, together with 95% credible intervals. The computed probabilities were then applied to the population of China aged 15 years and above in the corresponding years in both the rural and urban settings to derive the expected number of cases (“burden”). In this model, “point prevalence” refers to the probability that a randomly sampled individual from an urban or rural setting has schizophrenia in a given year (1990, 2000 or 2010), while “lifetime prevalence” refers to the probability that the same individual reported having been diagnosed with schizophrenia during his/her lifetime by the given year. The population size of China for 1990, 2000 and 2010 was obtained from the United Nations Population Division [11] while the proportion of urban residency for the corresponding years was obtained from census conducted by the National Bureau of Statistics of China [18].

To explore the effect of two other possible covariates – the distribution of subjects within each sample by sex and age – on which we did not have complete information from all studies, we conducted a separate sensitivity analysis using all the studies in our data set that provided information on age and gender of the examinees. We evaluated the effects of mean age of the sample and male–to–female ratio on the point and lifetime prevalence of schizophrenia using Bayesian methods described in eMethods in **Online Supplementary Document(Online Supplementary Document)**.

## RESULTS

Bayesian analyses of the 42 studies combined information from 2 284 957 people tested for schizophrenia, 10 506 of whom were diagnosed with the disease at some point in their lives. Analyses of the point prevalence data suggested that the increase in prevalence was significant in urban areas, but not in rural areas (eMethods in **Online Supplementary Document(Online Supplementary Document)**). This finding was corroborated with a separate analysis of the data on lifetime prevalence, which showed a significant and positive effect of the year of study in both the rural and the urban settings, confirming a significant increase in the prevalence of schizophrenia over the two decades.

Based on those results, it is possible to estimate the probabilities of having schizophrenia in 1990, 2000 and 2010 in urban and rural settings, together with a 95% credible interval. **Table 2** shows that, in urban areas of China, the point prevalence in the population aged 15 years or older was 0.32% (0.29–0.36%) in 1990, 0.47% (0.44–0.50%) in 2000 and 0.68% (0.57–0.81%) in 2010. In contrast, in rural areas, the corresponding probabilities were 0.37% (0.33–0.42%), 0.36% (0.35–0.38%), and 0.35% (0.33–0.38%). Lifetime prevalence in the population aged 15 years or older in urban China was 0.39% (0.37–0.41%) in 1990, 0.57% (0.55–0.59%) in 2000 and 0.83% (0.75–0.91%) in 2010. The corresponding probabilities for rural areas were 0.37% (0.34–0.40%), 0.43% (0.42–0.44%), and 0.50% (0.47–0.53%) (**Table 2**).

**Table 2:** Estimates of lifetime and point prevalence of schizophrenia in urban and rural settings in China for the year 1990, 2000 and 2010 (with 95% credible intervals)*

Outcome/Setting	Year		
	**1990**	**2000**	**2010**
**Point prevalence:**			
Urban	0.32% (0.29–0.36%)	0.47% (0.44–0.50%)	0.68% (0.57–0.81%)
Rural	0.37%(0.33–0.42%)	0.36% (0.35–0.38%)	0.35% (0.33–0.38%)
**Lifetime prevalence:**			
Urban	0.39% (0.37–0.41%)	0.57% (0.55–0.59%)	0.83% (0.75–0.91%)
Rural	0.37% (0.34–0.40%)	0.43% (0.42–0.44%)	0.50% (0.47–0.53%)
**Number of cases of disease (in thousands):**			
Urban	849 (805–892)	1935 (1867–2003)	4412 (3987–4837)
Rural	2245 (2063–2427)	2607 (2546–2667)	2744 (2580–2901)
All China	3094 (2868–3319)	4542 (4413–4670)	7156 (6566–7748)

*The estimates are based on Bayesian methods and represent the median of posterior distribution of probability of having schizophrenia in the year 1990, 2000 and 2010. Computation of the number of cases is based on estimates of lifetime prevalence.

**Figure 3:**
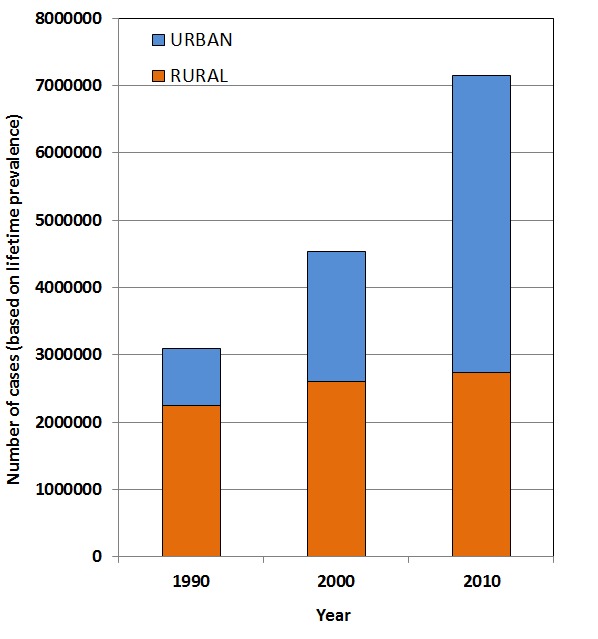
Absolute number of schizophrenia cases in China 1990–2010 by type of residency, based on estimates of lifetime prevalence.

Applying those probabilities to the corresponding population estimates for China and taking into account the percentage living in urban areas in 1990, 2000 and 2010, it is estimated that there were 3.09 (2.87–3.32) million persons in China affected with schizophrenia during their lifetime in the year 1990. 27% of the cases were from urban areas, which is comparable to the population of China rbanizatio as “urban” in 1990 in demographic records [11]. By 2010, the number of persons ever affected with schizophrenia has risen sharply to 7.16 (6.57–7.75) million – a 132% increase – while the total population of China only increased by 18% during this period [11]. Moreover, the contribution of expected cases from developed urban areas to the overall burden increased from 27% in 1990 to 62% in 2010, well above the proportion of urban residents in China in 2010 that was just below 50% (**Figure 3**) [11].

## DISCUSSION

Our study shows that in 1990, the probability that people would suffer from schizophrenia was very similar in rural and urban China. In urban areas, however, the prevalence rose steeply over the 20–year period, approximately doubling by 2010. We can be reasonably confident in our assessment of the prevalence and trend of schizophrenia cases in China over this period of rapid urbanization (1990–2010). This is in part because of the relatively large number of high quality studies that we were able to obtain from the Chinese databases. These studies gave us an unprecedentedly large overall sample size upon which our estimates were based. All studies included in our analyses were based on a consistent rural/urban separation that used the comparable case definition over the 20–year period [19–21]. Chance effects in our estimates were minimized because the studies were very large, spread over 21 of China’s 31 provinces, municipalities and autonomous regions (as shown in Table s4 in **Online Supplementary Document(Online Supplementary Document)**), applied comparable internationally recognized ‘gold standard’ case definitions of schizophrenia and used stringent data collection methods. Bayesian methods of analyses were suited to the study as they make good use of data sets based on very large sample size [22].

It is useful to compare our estimates with historic estimates that emerged from national surveys of psychiatric disorders that were performed in collaboration with the World Health Organization in 1982 and 1993 [23,24]. Based on those large multi–province efforts, Phillips et al. estimated that 4.25 million people in China were living with schizophrenia in the period 1995–1999 [24]; in another study they estimated 4.77 million cases in 1999 [23]. In our study, we estimated 4.54 (4.41–4.67) million cases in 2000, which is very similar to both of those previous estimates and falls between them. Moreover, Phillips et al. estimated that the prevalence of schizophrenia was higher in urban than rural areas (RR = 1.62 [1.10–2.40]), with exact values for 1993 of 6.71 per 1000 (59/8799) for the urban point prevalence, and 4.13 per 1000 (43/10424) for a rural point prevalence [24]. We conducted a similar analysis on a much larger sample derived from ten studies that explored the prevalence in both rural and urban population from the same geographic setting, using the same study design and methods of case ascertainment (see Table s5 in **Online Supplementary Document(Online Supplementary Document)**). We obtained estimates of 6.4 per 1000 (480/74 925) for urban and 4.4 per 1000 (342/77 529) for rural point prevalence. Again, those estimates are very similar and supportive of each other, providing additional validity to our findings [24,25].

In addition, biases in estimates are limited by the exclusion of studies with special populations, and by performing an additional sensitivity analysis for the effects of age and gender distribution within the samples on the reported prevalence. Through the latter, we were able to exclude the role of mean age as a significant predictor and to establish higher rates among males. Given that male–to–female ratio across all studies was comparable to the population of China, no further adjustments were required (eMethods in **Online Supplementary Document(Online Supplementary Document)**). The effect of development, industrialization and urbanization is unlikely to be partly explained by migrant workers from rural areas increasingly seeking care in urban areas. This is because rural prevalence has not decreased significantly over the same period and because previous studies provide further evidence that such scenario is unlikely [26,27]. Moreover, the possible explanation of affected persons selectively moving from rural to urban areas for treatment is not supported by what is known about care seeking by migrant groups [22,26].

Several further variables could have hypothetically contributed to the results. The first one is a possible difference in level of awareness about the symptoms between rural and urban areas. The second one is related to greater social and occupational demands of urban living vs rural living, that make the limitations and social dysfunction of individuals with schizophrenia more obvious in urban areas. Moreover, social networks may be better developed in rural areas, or attitudes towards psychiatric conditions more negative, resulting in a larger proportion of individuals with schizophrenia being missed, unreported, or refusing to participate in studies. Given that nearly two–thirds of the studies were conducted by the teams of investigators that visited both urban and rural areas, with case definitions applied strictly, we conclude that those potential biases should not be expected to have a major influence on the reported results.

Another hypothetical bias is a possibility of considerably higher fatality of persons with schizophrenia in rural areas, which would remove them from the pool of cases identified in prevalence studies. We found three studies that provided useful information to address this problem [24,28,29]. They all agreed that suicides are still relatively rare among the cases in both areas. Therefore, mortality and fatality rates reported in those studies are not sufficiently large to explain an appreciable portion of the observed differences in prevalence rates.

This study also helps address bias towards high–income countries in studies on the effects of development, industrialization and urbanization in the current literature on schizophrenia. It helps establish the universality of this risk factor and the extent to which it affects the burden of schizophrenia in a large country that underwent rapid urbanization. Such associations could not be have been explored in high–income countries because industrialization had occurred over a much longer period of time, with little reliable epidemiological records.

Nonetheless, there are a number of limitations. The large effect of development, industrialization and urbanization on the prevalence of schizophrenia in China does not imply causality. To this end, our study is able to shed some light on possible mechanisms through which development and urbanization increase the risk of schizophrenia, by lending support to some hypotheses over others. As schizophrenia prevalence was found to be similar in the beginning of this period of industrialization (late 1980s) in both rural and urban China [30,31], our findings suggest that the mechanisms driving the risks of illness in urban areas are likely to be associated with modern urban lifestyles and the development of urban areas. If urban birth or maternal exposure to infectious diseases (linked to higher population density in urban areas) were primarily responsible, then we would have expected notable differences between urban and rural areas across the entire study period, but this was not the case.

We are unable to further explore possible mechanisms due to the lack of information relating to the characteristics of individuals with and without schizophrenia in the original studies, such as their living conditions, degree of isolation, migration status, age of migration, time spent in urban areas, and population density of the individual’s place of original and the study sites. Due to the insufficient number of incidence studies identified in our review, we are unable to define the trend of incidence of schizophrenia in China over this period, which could have further informed the ways in which development, industrialization and urbanization affect the rates of schizophrenia. To fully understand these associations, further studies should focus on establishing large high quality and nationally representative cohort with information on different socio–demographic structure and individual characteristics [32,33]. The lower rates of schizophrenia found in the beginning of the study period (in 1990), when China was less industrialized, are consistent with previous studies that reported lower rates of schizophrenia in LMIC [3,4,10]. Previous studies also showed that those affected with schizophrenia in pre–industrialized regions seemed to have a better prognosis for recovery, regardless of a lack of treatment [34,35].

The “population attributable fraction” due to development, industrialization and urbanization cannot be computed in a standard way because this would require the relative risk to be constant over time. In our study, it appears that the relative risk of schizophrenia for urban area residency has been increasing over the past two decades, from being approximately 1.0 to 2.0 or higher. The relative risk of >2.0 is comparable to that reported in studies of urban areas in high–income countries [6,9]. This changing risk of urban area residency in China over the 20–year period makes it difficult to assign a particular proportion of schizophrenia cases to urban area residency in the year 2010. If we accept that the relative risk of urban area residency increased to 2.0 in 2010, then approximately 1.38 million out of 7.16 million cases would be attributable to a change of residency status for the 300 million Chinese whose rural area residency in 1990 changed to urban area residency by 2010. Another way of looking at this problem is to also include the urban residents in 1990 who were exposed to a much higher risk of urban living by 2010, suggesting that up to 3 million schizophrenia cases in 2010 are the “excess” attributable to living in modern, industrialized and developed urban areas (**Figure 3**). These computations are based on two components: (i) increased exposure to “urban area living” in 2010 (in comparison to 1990, one quarter of the population of China moved from “unexposed” rural to “exposed” urban environment); and (ii) increased prevalence in urban environments (with a rough estimate of RR of 2.0 in 2010). This implies that 19.3% of schizophrenia cases in China today may be due to transition from rural to urban environment, with a further 21.6% might be due to changes in lifestyle among people who were already living in urban environment in 1990.

This work has broad implications. Living in urban areas seems to be the most consistently identified environmental risk factor for schizophrenia in the English literature, with a substantial relative risk and population attributable fraction noted in western countries [6,9]. The relevant Chinese literature reviewed in this study provides additional evidence in support to this observation in a different ethnic population. Many populous parts of the world, particularly in LMIC, are undergoing development, industrialization and urbanization at a scale and rate that took western countries centuries to achieve [36]. Global development, industrialization and urbanization may therefore result in an increased global prevalence of schizophrenia through mechanisms that need to be further explored. Recently, a landmark study that investigated the genetic basis of schizophrenia was published, showing that “…*associations (between genetic variants and schizophrenia) were enriched among genes expressed in tissues that have important roles in immunity, providing support for the speculated link between the immune system and schizophrenia*” [37]. Given that improved sanitation and decreased exposure to infections are one of the main consequences of urbanization, this unexpected finding further underscores the importance of further epidemiological exploration of an apparent association between urbanization and schizophrenia.
