# Supplementary material for: Prevalence of schizophrenia in China between 1990 and 2010
Source: J Glob Health. 2015 Jun 20;5(1):010410. doi: 10.7189/jogh.05.010410 (PMC4663755; doi:10.7189/jogh.05.010410)
Supplement: Online Supplementary Document [file jogh-05-010410-s001.pdf]

Online Supplementary Document

Chen and Rudan et al. Prevalence of schizophrenia in China between 1990 and 2010

J Glob Health 2015;5:010410

Table s1: A detailed breakdown of the results of initial search of the Chinese databases for the papers on epidemiology of schizophrenia between 1990 and 2010. (note: R1=FFZ; R2=SJM)

Search Term		Search Term		Number of Titles				Relevant Titles				Relevant Abstracts			
				CNKI		WF		CNKI		WF		CNKI		WF	
				R1	R2	R1	R2	R1	R2	R1	R2	R1	R2	R1	R2
1. 精神分裂 (The Chinese term for schizophrenia `	+	发病率	(incidence rate)	686	686	208	208	17	22	5	2	17	22	4	2
		发生率	(incidence rate)	1590	1590	1182	1182	14	14	2	2	10	14	2	2
		患病率	(prevalence rate)	487	487	304	304	95	111	73	66	86	111	69	66
		流行	(epi*)	161	161	227	227	80	90	69	63	71	90	67	63
		死亡率	(mortality rate)	97	97	36	36	0	1	0	0	0	1	0	0
		病死率	(case fatality rate)	20	20	17	17	0	0	0	0	0	0	0	0
		现患率	(point prevalence rate)	18	18	5	5	5	5	2	3	3	5	2	3
		罹患率	(attack rate)	6	6	15	15	0	0	1	0	0	0	1	0
		现况调	(cross-sectional study)	69	69	20	20	30	30	4	4	28	30	3	4
		现况研	(cross-sectional study)	31	31	3	3	2	3	0	0	2	3	0	0
2. Schizophrenia	+	发病率	(incidence rate)	645	645	27	27	17	16	2	1	16	16	1	1
		发生率	(incidence rate)	1455	1455	472	472	12	13	0	0	9	13	0	0
		患病率	(prevalence rate)	427	427	40	40	62	77	14	13	54	77	12	13
		流行	(epi*)	128	128	44	44	57	67	11	13	51	67	11	13
		死亡率	(mortality rate)	65	65	2	2	0	0	0	0	0	0	0	0
		病死率	(case fatality rate)	18	18	1	1	0	0	0	0	0	0	0	0
		现患率	(point prevalence rate)	12	12	1	1	6	6	1	1	4	6	1	1
		罹患率	(attack rate)	5	5	1	1	0	0	0	0	0	0	0	0
		现况调	(cross-sectional study)	69	69	6	6	31	31	1	1	27	31	1	1
		现况研	(cross-sectional study)	42	42	0	0	2	3	0	0	2	3	0	0
Total				6031	6031	2611	2611	430	489	185	169	380	489	174	169

Table s2. The full list of studies retained for the analyses (Note: The list employs the official English translation of the journal names, journal abbreviations and paper titles as evident in the printed journal, CNKI, WanFang and other official academic databases. Wherever the official English translation of the journal names was not available, a pinyin title is used. (*indicates translation of Chinese paper titles by the authors of this manuscript for Chinese papers where official English translation is not available).

Study ID	References
Cross-sectional studies with information on the prevalence of schizophrenia (n=42)	
**Sc4**	Cheng CA, Ai CQ, Cheng JL, He JB, Ma GZ, He YM (成传安,艾春启,成加林,何君波,马国之,何咏明). Epidemiology investigation on mental disorder in Danjiangkou city in 1998 (丹江口市 1998 年精神病流行病学调查). *Chinese Journal of Civil Administration and Medicine (中国民政医学杂志)* 2001; 13: 334-5, 337.
**Sc6**	Huang JM, Lv JC, Xu JQ, Chen XQ, Huo BQ, Jiang LY, Zeng JS, Liu LH (黄劲梅,吕嘉春,许家锵,陈小青,霍宝琼,蒋丽仪,曾金生,刘丽华). A cross-section study and quality of life evaluation for mental disorders in a community (佛山市某社区精神病现况调查和生活质量测量). *Journal of Preventive Medicine Information (预防医学情报杂志)* 2001; 17: 420-2.
**Sc10**	Cui LJ, Li KQ, Cui Z, Jiang QP, Gao LH, Zhang Y, Li JF , Liu YQ, Sun XL, Han YC, Yang LH, Yan BP, Lv H, Yang BL, Yang YJ (崔利军,栗克清,崔泽, 江琴普, 高良会,张扬,李建峰,刘永桥,孙秀丽,韩延超,杨老虎,严保平,吕华,杨宝丽,杨怡静). Prevalence, demographic characteristics and function status of the schizophrenia in Hebei province (河北省精神分裂症的患病率、人口学特征及功能状况分析). *Chinese Journal of Nervous and Mental Diseases (中国神经精神疾病杂志)* 2007; 33: 155-8.
**Sc11**	Wang ZW, Chen FS, Sun PJ (王志文,陈福生,孙佩金). Analysis of an epidemiological survey of schizophrenia in the Suihua city of Heilongjiang province* (黑龙江省绥化市精神分裂症流行学调查分析). *Journal of Qiqihar Medical College(齐齐哈尔医学院学报)* 1992; 13: 183-7.
**Sc13**	Lu XY, Cheng HL, Hu B, Chen XS, Zou GH, Zhou PL, Li ZC, Wu SH, Kuang YH, Liu P, Liu ZY, Chen DH, Liu KF, Zhou GZ, Li CF, Zhu AX (卢小勇,陈贺龙,胡斌,陈宪生, 邹国华,周平良,李正春, 吴书华,匡奕华,刘平,刘增裕,陈点火,刘快发,周国治,李春芳,朱安雄). Epidemiological survey on prevalence of schizophrenia in Jiangxi province (江西省精神分裂症患病率流行病学调查). *Shanghai Archives of Psychiatry (上海精神医学)* 2004; 16: 234-6.
**Sc14**	Wan C, Fu MZ, Lan SZ, Zhu XG (万纯,付美珍,兰胜作,朱贤苟). Results of a prevalence study of Schizophrenia in Yichun city, Jiangxi province* (江西省宜春市精神分裂症患病率的调查结果). *Sichuan Mental Health (四川精神卫生)* 2002; 15: 178.

**Sc22**	Li CL, Shen CQ, Luo TK, Li ZX, Cheng PF (李成林,沈崇庆,罗大冲,李中祥,程鹤方). A genetic epidemiological investigation of schizophrenia in Supoxiang* (苏坡乡精神分裂症遗传流行学调查). *Journal of Clinical Psychological Medicine (临床精神医学杂志)* 1992; 2: 199-201, 213.
**Sc23**	Yuan GZ, Zhang ML, Wu XM, Liu YS, Tang RC, Yao JJ, Chu X, Qian YC, Ji Q, Zhang FJ, Qi SG, Xu WW, Zhang X, Liu XW, Huang YP, An BF, Zhou DX, Jiang XY, Bao ZH (袁国桢,张明廉, 吴晓梅,刘雨生,唐瑞春,姚建军,储兴, 钱永潮,季庆,张凤娟,祁曙光,徐文炜,张霞,刘晓伟,黄寅平,安宝富,周德祥, 蒋幸衍,包炤华). Prevalence rate and related factors of schizophrenia among 11940 subjects in the urban and rural areas of Wuxi city (无锡市 11940 名城乡居民精神分裂症患病率及其相关因素分析). *Chinese Journal of Clinical Rehabilitation (中国临床康复)* 2004; 8: 7366-7.
**Sc26**	De-Chang Chen,Jing-Ai Feng(陈德昌,封敬爱). An epidemiological study on schizophrenia-related disabilities in Zibo city*. (淄博市精神分裂症残疾的流行病学调查). *Shandong Mental Health (山东精神医学)* 1996; 1: 7-9.
**Sc27**	Guo HL, Zhu ZH, Huang LY(郭红利,朱振华,黄良衍). Present and future state of mental health of Beijing in 1990s - An epidemiologic investigation of mental disorders in Beijing in1991* (90 年代首都精神卫生间题的现况与前瞻——北京市 1991 年精神障碍流行学调查报告). *Journal of Clinical Psychiatry(临床精神医学杂志)* 1994; 4: 131-4.
**Sc30**	Chen YC,Zhang WX, Li SR, Chen CH,Luo KL(沈渔邨,张维熙,李淑然,陈昌惠,罗开林). An epidemiological survey of mental disorders, mental health services and cognitive impairments in seven regions of China in 1997* (1997 年中国 7 地区精神疾病 、精神卫健服务和精神与智力残疾流行病学调查). *Bulletin of Medical Research(医学研究通讯)* 2000; 29: 14-5.
**Sc33**	Wang SJ, Wei ZY, Niu F, Zhang K, Wei XB, Zhang KM, Cheng XF, Xi XY, Wu QY, Liu Q, Ning NY, Yu SJ, Gao K, Liu XG, Guo H, Guo CL, Liu XH, Cai HY, Peng XF (王世纪,韦志岩,牛飞,张岿,韦学斌,张克民,程效芬,奚雪英,吴启英,刘青,宁南义,于世杰,高坤,刘恒芬,张新功,郭虹,郭翠玲,刘晓红,蔡贺云,彭学富). An epidemiological survey on schizophrenia in Fuyang, Anhui (安徽阜阳市精神分裂症流行病学调查). *Journal of Clinical Psychological Medicine (临床精神医学杂志)* 2001; 12: 3-4.
**Sc38**	Xiao QX,Zheng ZH, Wu BH (肖秋霞,郑志华,吴宝恒).Epidemiology survey of mental disorders in Daxing district of Beijing in 2004 (北京市大兴区 2004 年精神障碍流行病学调查报告). *Chinese General Practice (中国全科医学)* 2007; 10:1216-8.
**Sc42**	Meng QZ, Yu ZG, Liu JP, He PH, Zhang XP, Liu J, Ma HZ, He PE (孟庆珠,余志刚,刘金鹏,何培惠,张新平,刘军,马恒州,何培恩). An epidemiological investigation of mental disorders in rural populations* (对农村人群精神疾病流行病学及相关情况调查). *Medical Journal of Chinese Civil Administraion(中国民政医学杂志)* 2001; 13: 31-2.
**Sc47**	Hu JM, Li Z, Chen YH, Zhou XM Ma YX, Huang HF, Yan HR, Wang XL, Guan LY, Wang WB (胡季明,李 真,陈贻华,周湘梅,马宇行,黄海峰,严惠然,王向林,关莲英,王文波). Epidemiological investigation on schizophrenia in the city of Zhongshan, Guangdong (广东中山市精神分裂症流行病学调

	查). *Medical Journal of Chinese People Health (中国民康医学杂志)* 2003; 15:355-6, 336.
**Sc49**	Feng QM, Wei B,Chen Q, Pan RD, Chen QM, Huang GG, Su L, Chen FQ (冯启明,韦波,陈强,潘润德,陈秋明,黄国光,苏莉,陈发钦). Mental disorders among minority people in rural areas of Guangxi Zhuang Autonomous Region (广西农村少数民族居民精神疾病流行病学分析). *Chinese Journal of Public Health (中国公共卫生)* 2011; 27: 408-10.
**Sc50**	Gan JX, Xiao X, Huang HB, Li J (甘记兴,肖信,黄海彬,李杰). An epidemiological investigation of mental disorders in Yulin city, Guangxi* (广西玉林市精神疾病流行病学调查). *Medical Journal of Chinese People's Health (中国民康医学)* 2008; 20: 2005-6.
**Sc52**	Wei B,Chen Q, Feng QM, Pan RD, Chen QM, Huang GG, Tang HN, Su L, Chen Y, Chen FQ, Li HJ (韦波,陈强,冯启明,潘润德,陈秋明,黄国光,唐峥华,唐海宁,苏莉, 陈娜萦,陈发钦,黎火佳). Epidemiological survey on mental disorders in urban and rural in Guangxi Zhuang autonomous region, China (广西壮族自治区城乡居民精神疾病流行病学调查). *Journal of Guangxi Medical University (广西医科大学)* 2010; 27: 951-6.
	Chen Q, Wei B, Feng QM, Pan RD, Huang GG, Chen QM, Su L, Tang ZH, Tang HN, Chen NY, Chen FQ, Li HJ (陈强,韦波,冯启明,潘润德,黄国光,陈秋明,苏莉,唐峥华,唐海宁,陈娜萦,陈发钦,黎火佳). Epidemiological survey of mental disorders in urban areas in Guangxi Zhuang autonomous region, China (广西壮族自治区城市居民精神疾病流行病学查). *Chinese Journal of Nervous and Mental Diseases (中国神经精神疾病杂志)* 2010; 36: 458-62.
	Wei B, Feng QM, Chen Q, Pan RD,Chen QM, Huang GG, Tang ZH, Tang HN, Su L, Chen NY, Chen FQ, Li HJ (韦波,冯启明,陈强,潘润德,陈秋明,黄国光,唐峥华,唐海宁,苏莉,陈娜萦,陈发钦,黎火佳). Epidemiological survey on mental disorders in rural in Guangxi Zhuang autonomous region (广西壮族自治区农村地区精神疾病流行病学调查). *Modern Preventive Medicine (现代预防医学)* 2011; 38:1801-5.
**Sc54**	Zhao ZH, Huang YQ, Li J, Deng HH, Huang XM, Su JH, Dang WM, Yang Y, Huang JK, Zhang WM, Deng Y, Zhou WC, Qiu C, Lu WC, Chen YW, Zhong SJ, Chen BY, Zeng QM, Mei F (赵振环,黄悦勤,李洁,邓河晃,黄杏梅,苏敬华,党卫民,杨翌,黄靖康,张卫敏,邓妍,周伟成,邱畅,卢玮聪,陈宇薇,钟思俊,陈碧媛,曾俏梅,梅芳). An epidemiological survey of mental disorders in Guangzhou area (广州地区常住人口精神障碍的患病率调查). *Chinese Journal of Nervous and Mental Diseases (中国神经精神疾病杂志)* 2009; 35: 530-4
**Sc55**	Mo XM, Liang WP, Chen JR (莫秀梅,梁卫萍,陈建荣) An epidemiological survey of mental disorders in Liwan street, Hualin district, Guangzhou city* (广州市荔湾区华林街精神病患病情况调查分析). *South China Journal of Preventive Medicine (华南预防医学);*2003, 29(1):48-49
**Sc58**	Zhang DR, Jia LC, Zhou C,Wang LQ, Wu AR, Wu G, Luo HY, Rong DS, Huang XC, Cao YM, Su H, Zhang W (张迪然,贾良春,周曹,王列全,吴安仁,吴刚,罗环跃,戎笛生,黄信初,曹玉鸣,粟宏,张薇). A comparative study on the epidemiological investigation of mental disorders in some areas of Guizhou province (贵州省部分地区精神疾病流行病学调查比较研究). *Journal of Guiyang Medical College (贵阳医学院学报)* 2003; 28: 515-8.

**Sc61**	Ye TW, Wu CD, Chen WX, Wang SY, Cai YD, Yang ZQ, Yu MH, Han TM, Luo HD, Li LC, Huang S, Huang JD, Wang GL, Xie YN, Xu ZX, Wu TG, Guan SM, Zhu GQ, Wu CE (叶廷蔚,吴传东,陈维雄,王世云,蔡有德,杨志清,余明豪,韩天明,罗海东,李连昌,黄胜,黄进弟,王桂莲,谢有浓,许正雄,吴挺刚,官尚民,朱国钦,吴承泽) An epidemiological investigation of mental disorders in Hainan province* (海南省精神疾病流行病学抽样调查). *Hainan Medical Journal (海南医学)* 1995; 16: 257-60.
**Sc62**	Chen WX, Wu CD, Wu CZ, Wang GL, Xie YN, Li LC, Ye TW, Wang SY, Luo HD, Yang ZQ, Yu MH, Han TM(陈维雄,吴传东,吴承泽,王桂莲,谢有农,李连昌,叶廷蔚,王世云,罗海东,杨志清,余明豪,韩天明). An epidemiological survey of mental disorders amongst Han and Li ethnic groups* (汉黎族精神疾病流行学调查). *Journal of Health Psychology* (健康心理学)* 1996; 4:186-8.
**Sc74**	Shen JX, Zhao ZS (沈锦相,赵振声). Epidemiological survey on the handicap caused by schizophrenic psychosis(精神分裂症残疾的流行病学调查). *Journal of Dali Medical College (大理医学院学报)* 1997; 6: 34-6.
**Sc75**	Zhang WL (张尉良). An epidemiological survey of disabilities caused by schizophrenia* (精神分裂症残疾的流行病学调查). *Journal of Clinical Psychiatry (临床精神医学杂志)* 1993; 3: 198-9.
**Sc76**	Zhang JX, Weng Z (张敬悬,翁正). An investigation of mental disability in schizophrenia and its risk factors related (精神分裂症的精神残疾及其相关因素的调查分析). *Sichuan Mental Health (四川精神卫生)* 2001; 14: 69-71.
**Sc83**	Tan J, Yu XQ, Han ZQ, Chen TJ,Yu SC, Deng J, Wang DW, Jiang GH, Ye WL, Yu Y, Lei ZC, Li XQ (谭剑,余晓琼,韩志琴,陈体君,余树成,邓杰,王德伟,江光华,叶文莉,余 颖,雷震川,李雪群). An epidemiological study on the natural history of schizophrenia in rural areas* (农村精神分裂症自然病程流行病学调查). *Journal of Clinical Psychosomatic Diseases (临床心身疾病杂)* 2009; 15: 156-7.
**Sc85**	Zhan RT, Lu WX, Li SZ, Xiang XH, Chen WM, Li T, Xiao JM (詹荣庭,鲁文兴,黎世泽,向兴华,陈文明,李涛,肖建梅). An epidemiological survey of mental disorders in Pu'er city* (普洱市精神疾病现状调查). *Medical Journal of Chinese People's Health* (中国民康学)* 2011; 23: 955,1012.
**Sc90**	Zhang XG, Zhuang XH, Chen JF, Zhao DQ (张献共,庄希航,陈静芳,赵丹青). Epidemiological study of schizophrenia in metropolitan Shantou (汕头市精神分裂症流行病学调查). *Journal of Clinical Psychological Medicine (临床精神医学杂志)* 1997; 7: 83-5.
**Sc91**	Xu Q, Chi S, Luo XW, Dai D,Lu YC, Ding GQ,Yan FJ, Li QR, Gui HM, Liu JF, Ye XY, Lin CF, Liu XH (徐琼,池森,罗贤文,戴迪,卢银财,丁国庆,颜福娟,黎庆荣,归红敏,刘家丰,叶小玉,林彩凤,刘兴华). Epidemiological survey on mental disorders in Shangrao (上饶市精神疾病流行病学调查). *Shanghai Archives of Psychiatry (上海精神医学)* 2003; 15: 225-7.
**Sc92a**	Zhang JH, He WM, Yan WL, Gan JG, Shi YY (章金辉,何为民,严伟良,甘建光,石永扬). Study on the prevalence of mental diseases in Shaoxing city between 1991 and 2001(绍兴市 1991 年与 2001 年精神疾病患病率调查). *Chinese Journal of Epidmiology (中华流行病学杂志)* 2003; 24:

	585-7.
**Sc93**	Chen YQ, Shen DY (陈怡强,沈调英). Epidemiological investigation on mental disorders in Shaoxing county (绍兴县社区人群精神疾病流行病学调查). *Strait Journal of Preventive Medicine (海峡预防医学杂志)* 2004; 10: 7-9.
**Sc98**	Deng XF, Yang MH, Zou JH, Peng HZ, Du JL, Zhou XH, Gao HJ,Tang ZH, Liu YQ, Zhao DM, Gu WJ, Wu B,Zhu YQ, Ding YJ(邓晓舫,杨明辉,邹建华,彭虹珍,杜俊林,周秀华,高红君,唐中华,刘玉全,赵冬梅,古伟敬,吴斌,朱永强,丁永军). Epidemiological survey of the mental illness in Leshan city of Sichuan province (四川省乐山市精神疾病流行病学调查). *Medical Journal of Chinese People Health (中国民康医学杂志)* 2003; 15:265-8, 271.
**Sc100**	Dong AL, Zhang K, Wang SS, Sun JG, Zhang LX, Zhang CH,Gong ZY, Tang SJ,Bi JH,Gu DH,Zhang XR,Niu N,Mu JM,Cui SK (董爱玲,张岿,王善松,孙家果,张立贤,张翠红,宫兆瑛,唐松军,毕见好,谷旦华,张秀茹,牛娜,母金明,崔世考). Epidemiological survey of mental disorders in Weihai (威海市精神疾病流行病学调查). *Journal of Clinical Psychological Medicine (临床精神医学杂志)* 2008; 18: 241-3.
**Sc101**	Gao ZZ, Cao XY, Zhu JP, Zhu GH, Gao WB, Kong YY,Niu GJ,Si GM, Sun YJ, Li N, Wang QG, Guo H, Wei QL, Xu DJ, Song JZ, Bao L, Zheng WQ(高振忠,曹学义,朱建平,朱国辉,高伟博,孔媛媛,牛国静,司桂梅,孙亚杰,李娜,王秋刚,郭宏,魏启龙,徐德军,宋建芝,包丽,郑潍清). The 3rd epidemiological study of mental disorders in Weifang* (潍坊市第三次精神障碍流行病学调查). *Chinese Journal of Nervous and Mental Diseases (中国神经精神疾病杂志)* 2006; 32: 152-3.
**Sc103**	Cao XY, Zhu JP, Tian GS, Wang WL, Yu GD, Wang XQ(曹学义,朱建平,田光昇,王维良,于观斗,王熙庆). A ten-years **case control** epidemiology study of mental disorders in Weifang* (潍坊市精神疾病流行学十年对照研究). *Shandong Archives of Psychiatry (山东精神医学)* 1997; 3: 19-22.
**Sc106**	Wei G, Liu SM, Zhang W,Xiang Y,Huang XQ,Yang C, Huang WJ, Xie WJ, He X, Su XF, Wang J, Ciren PC, Baima ZG, Zha S, Liu XH(魏赓,刘善明,张伟,向云,黄晓琦,杨闯,黄文君,谢维爵,何侠,苏晓凡,旺加,次仁平措,白玛卓嘎,次普,扎桑,刘协和). Epidemiological investigation on mental disorders at Tibet in China I: major psychiatric disorders (西藏自治区精神障碍流行病学调查 I:重型精神障碍). *Chinese Journal of Nervous and Mental Diseases (中国神经精神疾病杂志)* 2008; 34: 601-3.
**Sc109**	Sheng JL, Bai SZ, Zhao L, Cai DX, Wang YM, Wang AJ, Wang YY (盛嘉玲,白淑芝,赵灵,蔡德祥,王益民,王爱军,王友谊). An epidemiological survey of mental disorders in the Xinshi district of Urumqi city, Xinjiang autonomous region* (新疆乌鲁木齐新市区精神疾病流行病学调查). *Medical Journal of Chinese Civil Administration (中国民政医学杂志)* 2000; 12(1) :33-35.
**Sc110**	Ran MS,Xiang MZ, Li SX, Shan YH, Huang MS, Duan CF, Li SG, Liu ZR, Wan Y (冉茂盛,向孟泽,李胜先,单友荷,黄明生,段诚凤,李斯干,刘宗仁,万云). An epidemiological study of schizophrenia in the rural community of Xinjin County* (新津县农村社区精神分裂症流行病学调查). *Chinese Journal of Psychiatry. (中华精神科杂志)* 1998, 31: 123.

**Sc111**	Ma ZW, Li MX, Han Y, Wang ZM, Niu JL, Zhao L, Shi L, Wang XQ, Li CX, Wang W, Meng QW, Zhang XD (马振武,李梅香,韩嫣,王志敏,牛军力,赵琳,史丽,王新权,李彩霞,王伟,孟庆伟,张小栋). Epidemiological investigation on mental disorders in XinXiang city (新乡市精神疾病流行病学调查). *Medical Journal of Chinese People's Health (中国民康医学杂志)* 2005; 17: 501-2.
**Sc112**	Yang XB, Yang J, Liu XY, Duan XY, Xu XF, Yang JZ (杨晓斌,杨军,李秋媛,段馨懿,许秀峰,杨建中). The epidemiological investigation of schizophrenia in Hani people in Yuanyang (南元阳县哈尼族精神分裂症的流行病学调查). *Journal of Kunming Medical University (昆明医学院学报)* 2009; 30: 1-3.
**Sc118**	Xiang YT, Ma X, Cai ZJ, Li SR, Xiang YQ, Guo HL, Hou YZ, Li ZB, Li ZJ, Tao YF, Dang WM, Wu XM, Deng J, Lai KY, Ungvari GS. Prevalence and socio-demographic correlates of schizophrenia in Beijing, China. *Schizophrenia Research* 2008; 102: 270-7.

Table s3. The full list of studies retained for the analyses (Note: The list employs the official English translation of the journal names, journal abbreviations and paper titles as evident in the printed journal, CNKI, WanFang and other official academic databases.

Study ID	Population, setting and median year of study	Sampling	Diagnostic instruments and/or tools	Assessors and case finding method	Sample size, number of positively identified cases and age range	Agreement between assessors and quality control measures
**Sc4**	Residents of Danjiangkou city (1998)	Random cluster sampling (every sampling point investigated 600 households)	1.Chinese Classification of Mental Diseases-2-R (CCMD-2-R)<br>2. Additional tools (the manual of psychopathy epidemiology survey)	1. Phase 1: village doctors<br>2. Phase 2: three psychiatrists with more than 5 years clinical experience	6,486 (sample);<br>35 (lifetime);<br>28 (point);<br>age ≥15	1. Phase 1: trained for one week before the survey (Kappa=0·81)<br>2. Phase 2: trained for 2 weeks before the survey (ICC=0·93-0·97).
**Sc6**	Residents of Zumiao street in Foshan city (1998)	Random sampling based on a census data	1. CCMD-2<br>2. Additional tools:<br>a. Screening scale of mental disease by 10 questions;<br>b. Screening scale of neurosis by 12 questions;<br>c. Screening scale of children intelligence by 40 questions;<br>d. Social Disability Screening Schedule (SDSS);<br>e. Present State Examination (PSE);<br>f. Scale for the assessment of Negative Symptoms;<br>g. Mental handicap rating scale for adults;<br>h. Wechsler intelligence scale for Children (WISC);<br>i. COOP/WONCA questionnaire;<br>j. Activity of daily living scale (ADL);<br>k. QL-index;	1. Phase 1: medical staff<br>2. Phase 2: psychiatrists at a psychiatric hospital	83,301 (sample);<br>312 (lifetime);<br>age ≥18	1. Phase 1: trained before the survey<br>2. Phase 2: trained before the survey

			l. Karnofsky Performance Status scale (KPS);<br>m. Definition and classification standard of psychiatric disability			
**Sc10**	Residents of 40 towns and 147 villages (2004)	Stratified multistage cluster sampling	1. Diagnostic and Statistical Manual of Mental Disorders-IV (DSM-IV);<br>2. Additional tools:<br>a. General Health Questionnaire with increased Mental Health Problems;<br>b. Structured Clinical Interview for DSM-IV-TR Axis l Disorders Patient Edition (SCID-I/P);<br>c. Global Assessment function (GAF)	42 doctors and 27 nurses from above city level psychiatric hospital	20,716 (sample);<br>130 (lifetime);<br>106 (point);<br>age ≥18	Trained for 4 weeks before the survey (Kappa=0.88)
**Sc11**	Residents of Suihua city (1990)	Not specified	1. CCMD-2;<br>2. International Classification of Diseases-9 (ICD-9).	Not specified in the methods - the results imply psychiatrist's visits	11,088 (sample);<br>71 (lifetime);<br>64 (point);<br>age ≥15	Not Specified
**Sc13**	Residents of Jiangxi province (2002)	Random stratified and cluster sampling based on elementary administrative units; after sampling, family households were selected, rather than collective	1. ICD-10<br>2. Additional tools:<br>a. Composite International Diagnostic Interview, (CIDI);<br>b. SDSS;<br>c. Social-demographic Inventory;<br>d. Schizophrenia history form (self-made)	Psychiatrists	15,939 (sample);<br>124 (lifetime);<br>92 (point);<br>age ≥15	Trained for 2 weeks before the survey (Kappa=0·67-1·00).

		households				
**Sc14**	Residents of Yichun city (1999)	Random stratified and cluster sampling	1. CCMD-2-R;<br>2. Additional tools:<br>a. Recording sheet of schizophrenic history<br>b. 10 questions screening form of mental illness<br>c. PSE;<br>d. Scale for the Assessment of Negative Symptoms<br>e. SDSS	The study combined clues-led case identification (100% retrieval rate) with door-to-door survey (86% response rate); diagnoses were confirmed by two physicians.	17,847 (sample);<br>116 (lifetime);<br>95 (point);<br>age ≥15	Trained before the survey (Kappa=0·76); randomly selected 10% households to determine false negative rate (0·0002). Case review showed no false-positive patients. Survey response rate 86%.
**Sc22**	Residents of Su Po village in western suburbs of Chengdu (1988)	Random stratified sampling	1. Diagnostic criteria for schizophrenia in DSM-III-R<br>2. Additional tools:<br>a. Clinical diagnostic criteria for schizophrenia revised by Chinese Medical Association in October, 1984;<br>b. Household socio-demographic registration form;<br>c. Mental health screening form;<br>d. Psychiatric history questionnaire;<br>e. PSE-65;<br>f. Family questionnaire.	A investigation team consisted of three psychiatrists and two physicians	7,843 (sample);<br>48 (lifetime);<br>41 (point);<br>age ≥15	Trained before survey (Kappa=0·77-0·79); at the end of investigation, randomly selected two communities for review, with consistency rate of ICC=99% (in 388 people, accounting for 3·6% of the sample).
**Sc23**	Residents of Wuxi City (5 survey sites respectively in urban and rural areas) (2003)	Multistage random cluster sampling	1. CCMD-3<br>2 Additional tools:<br>a. Mental health screening form;<br>b. PSE;<br>c. SDSS;<br>d. Scale for assessment of negative symptoms (SANS);<br>e. Adult mental disability rating scales;<br>f. Definition and standard of mental disability;	A group of 40 medical personnel with more than 5 years of psychiatric clinical experience (6 with senior professional title, 14 with middle professional title, 20	11,940 (sample);<br>60 (lifetime);<br>48 (point);<br>age ≥15	Trained before survey (Kappa=0·68-0·86); reviewed 10% of negative subjects, found no false negatives.

			g. Household socio-demographic registration form;<br>h. Recording sheet of schizophrenic history	with primary professional title).		
**Sc26**	Residents of 60 villages in 6 towns and townships of Zibo city (1994)	Stratified unequal proportion random cluster sampling	1. CCMD-2<br>2. Additional tools:<br>a. Mental health screening form;<br>b. Mental health survey form;<br>c. PSE;<br>d. SDSS	A group of 9 psychiatric professionals conducted household survey that was facilitated by consulting "key informants" in the population.	8,616 (sample);<br>49 (lifetime);<br>age ≥15	Unified study and training before survey; ICC=95% for PSE and SDSS instruments; reviewed 10% of negative subjects, found no false negatives
**Sc27**	Residents of sixteen counties and districts (1991)	Random stratified and cluster sampling	1. Diagnostic and Statistical Manual of Mental Disorders-3, revised edition (DSM-III-R);<br>2. Additional tools:<br>a. CCMD revised, 1984;<br>b. The Operational Clinical Diagnostic Criteria of Neuroses in 1985;<br>c. The manual of mental diseases epidemiology survey (made by 12 organizations in China);<br>d. Adult mental disability rating scales (AMDRS);<br>e. Mini-Mental State Examination (MMSE);<br>f. Filtration table of drinking situation	141 investigators from 16 cooperation units; 90.78% of the investigators were psychiatric professionals, 25.54% were senior physicians.	35,385 (sample);<br>255 (lifetime);<br>218 (point);<br>age ≥15	All the members took centralized off-job training (Kappa=0·82-0·93); reviewed 10% of negative subjects, found no false negatives; response rate for door-to-door survey was 83.4%
**Sc30**	Residents of seven different districts (1993)	Not specified	1. ICD-9<br>2. Additional tools:<br>a. The Manual of Psychiatric Epidemiological Survey, 1985;<br>b. PSE and other tools;<br>c. SDSS	Not specified in methods, but referred to the methodology of the first multi-province survey in 1982 that involved two-stage case identification by trained investigators (Stage	19,223 (sample);<br>126 (lifetime);<br>102 (point);<br>age ≥15	Not specified; referred to the first survey in 1982

				1) and then psychiatrists (Stage 2).		
**Sc33**	Residents of urban area in Fuyang city and three counties (Yingshang county, Taihe county and Linquan county) (2001)	Stratified random sampling	1. CCMD-2-R<br>2. Additional tools:<br>a. Psychological health screening scale;<br>b. PSE;<br>c. Schizophrenia medical history questionnaire (self-made);	Not specified in methods, but results suggest a two-stage case identification by trained investigators (Stage 1) and then psychiatrists (Stage 2).	33,332 (sample);<br>159 (lifetime);<br>137 (point);<br>age ≥15	Trained before survey (Kappa=0·76~0·98)
**Sc38**	Residents of Daxing district (2004)	Random sampling based on census	1. ICD-10<br>2. Additional tools:<br>a. The manual of mental diseases epidemiology survey (made by 12 organizations in China);<br>b. Psychological health screening scale;<br>c. PSE;<br>d. SDSS;<br>e. Life event scale (LES);<br>f. AMDRS	6 psychiatrists divided into 2 groups	368,026 (sample);<br>2,032 (lifetime);<br>1,186 (point);<br>age ≥15	Trained before survey and carried out pre-survey in 50 households to assess false-negative rate; all team members underwent a rigorous professional training
**Sc42**	Residents of 19 villages in Beigou county in Xinyi city (1998)	Random sampling based on census	1. CCMD-2-R<br>2. Additional tools:<br>a. Various types of mental diseases screening scales;<br>b. Mental diseases patient related information questionnaire;	1. Phase 1: household survey by trained investigators;<br>2. Phase 2: suspected cases were referred to psychiatrists for diagnostic confirmation	35,757 (sample);<br>122 (lifetime);<br>age ≥15	Trained before survey (Kappa=0·60-1·00).

**Sc47**	Residents of Shiqi district in Zhongshan city and four towns (Minzhong town, Tanzhou town, Banfu town and Dayong town) (2000)	Multi-stage cluster random sampling (urban areas); economic stratified random sampling (rural areas)	1. CCMD-2-R<br>2. Additional tools:<br>a. Manual of mental diseases epidemiology survey, 1985;<br>b. Mental health screening scale;<br>c. SDSS;<br>d. Scale for the assessment of negative symptoms;<br>e. AMDRS;<br>f. Social-demographic information registration forms;<br>g. Mental history registration form of schizophrenia;<br>h. The definitions and grading standards of mental disability	Household survey conducted by trained investigators	2,909 (sample);<br>22 (lifetime);<br>20 (point);<br>age ≥15	Trained before survey; consistency test in pre-term and medium-term (ICC=95·0%-97·2%); reviewed 10% of sample to assess false-negative rate, found no false negatives
**Sc49**	Residents of 5 ethnic minority counties in Guangxi: Rongshui Miao, Sanjiang Dong, Jinxiu Yao, Luocheng Mulao and Huanjiang Maonan autonomous counties (2007)	Multi-stage cluster random sampling	1. ICD-10<br>2. Additional tools:<br>a. CIDI-3;<br>b. Socio-demographic characteristics questionnaire	1. Phase 1:<br>Each investigation team was made up of two psychiatrists and 12 field investigators - third year medical students from Guangxi Medical University who were familiar with the local dialects<br>2. Phase 2:<br>5% of those who screened negative to and 100% of those who screened positive were reviewed by psychiatric specialists using ICD-10.	4,743 (sample);<br>38 (lifetime);<br>31 (point);<br>age ≥15	1. All investigators were trained (Kappa=0·85-0·97);<br>2. All psychiatrists were trained according to ICD-10 (Kappa=0·91-1·00).<br>3. Data were double entered using EpiData 3·1.

**Sc50**	Residents of Yulin city (2007)	Stratified cluster probability proportional random sampling method (urban area : rural area=1:2)	1. ICD-10<br>2. Additional tools:<br>a. Household investigation scale;<br>b. The screening scale of mental illness Epidemiological survey;<br>c. CIDI-3;	A team of two psychiatrists with more than five years clinical experience, one postgraduate and nine undergraduate medical students	3,443 (sample);<br>23 (point);<br>age ≥15	All psychiatric clinicians were trained for 4 weeks; investigators trained for mental illness screening scales for 1 week (Kappa>70%)
**Sc52**	Residents of urban and rural areas in Zhuang autonomous region (included 6 prefecture-level cities and 10 counties) (2007)	Multi-stage random cluster sampling	1. ICD-10<br>2. Additional tools:<br>a. Household investigation scale;<br>b. CIDI-3;<br>c. Socio-demographic characteristics questionnaire (self-designed)	A team of 5 universities based, 2 mental health specialists, 12 practicing psychiatrists with more than five years of clinical experience, and 72 clinical medical undergraduate students.	18,219 (sample);<br>178 (lifetime);<br>151 (point);<br>age ≥15	All survey tools were translated into standardized minority languages; all non-psychiatrist investigators were trained for 1-2 weeks (Kappa=0·85~0v96); all psychiatrists were trained according to ICD-10 (Kappa=0·91-1·00)
**Sc54**	Residents of Guangzhou city (2006)	Stratified and cluster random sampling	1. DSM-IV-TR;<br>2. Additional tools:<br>a. CIDI-3·0 (Chinese version);<br>b. SCID-I/P (Axis I Disorders-Patient Edition)	Diagnosis by psychiatrists	7,418 (sample);<br>41 (lifetime);<br>age ≥15	Pre-survey training (Kappa≥0·75); 5% of negative cases (331 cases) were reviewed by an expert-working group made up of three senior psychiatrists. No false negatives
**Sc55**	Residents of Hualinjie	Informant clue-based	1. CCMD-3;<br>2. Handbook of prevention and rehabilitation of	The investigation team were	56,736 (sample);	Not specified

	community, Ligang district, Guangzhou (1998)	sampling using census	mental diseases;<br>3. Registration forms of prevention and rehabilitation and of mental diseases	consisted of psychiatrists and community mental health doctors. The door-to-door survey was facilitated by "key informants" from the population.	253 (point);<br>age ≥15	
**Sc58**	Residents of Guiyang city, Kaili city and Douyun city (2001)	Cluster random sampling	1. CCMD-3;<br>2. Additional tools:<br>a. The screening scale of mental disease, 10 questions;<br>b. The screening scale of neurosis, 12 questions;<br>c. The screening scale of children intelligence, 40 questions;<br>d. SDSS;<br>e. PSE;<br>f. AMDRS;<br>g. Wechsler intelligence scale for Children (WISC);<br>h. Scale for the assessment of negative symptoms;<br>i. Manual of Psychiatric Epidemiological Survey, 1985	Diagnosis by psychiatrists.	7,970 (sample);<br>30 (lifetime);<br>26 (point);<br>age ≥15	Pre-survey training (Kappa=85%-92%); review of 5% of households negative for cases, found no false negatives; reviewed all positive cases, found no false-positives
**Sc61**	Residents of Hainan province, (1994)	Stratified random sampling by ethnic, geographical and economic variables	1. DSM-III-R;<br>2. CCMD-2;<br>3. Manual of mental diseases epidemiology survey, 1985;<br>4. Registration form of household and alcohol screening	A team of 17 psychiatrists (including five attending physicians, two deputy chief physicians and one chief physician)	19,322 (sample);<br>98 (lifetime);<br>81 (point);<br>age ≥15	Pre-survey training for 3 weeks (ICC≥85%, Kappa≥0·65); pilot survey carried out before field investigation
**Sc62**	Residents of 30 villages in Tongshi (10 Han nationality; 20 Li	Mechanical stratified cluster sampling	1. CCMD-2<br>2. Manual of mental diseases epidemiology survey, 1985	Psychiatrists (one chief physician and three physicians)		Pre-survey training for 3 weeks (ICC≥85%; Kappa≥0·65)

	nationality) (1994)					
**Sc74**	Residents of DaLi prefecture, (1995)	Stratified and cluster random sampling	1. CCMD-2<br>2. Additional tools:<br>a. Manual of mental diseases epidemiology survey (developed by 12 organizations in China);<br>b. Mental health screening scale;<br>c. PSE;<br>d. SDSS	A team of seven psychiatrists.	6,088 (sample); 39 (lifetime); age ≥15	Pre-survey training (Kappa> 85%); 5% of the sample was reviewed for false negative rate, found no false-negatives
**Sc75**	Residents of Changzhou City (1991)	Stratified and cluster random sampling	1. CCMD-2<br>2. Additional tools:<br>a. The manual of mental diseases epidemiology survey (developed by 12 organizations in China);<br>b. Mental health screening scale;<br>c. PSE;<br>d. SDSS	A team comprising eight psychiatrists. A door-to-door survey facilitated by "key informants" in the population, where the Mental Health Screening Schedule was used.	4,708 (sample); 28 (lifetime); age ≥15	Pre-survey training (Kappa> 85%); review of 10 % of negative subjects, found no false-negatives
**Sc76**	Residents of 15 districts of the province (1994)	Stratified and cluster random sampling	1. CCMD-2<br>2. Additional tools:<br>a. PSE;<br>b. SANS;<br>c. SDSS	Trained investigators using scores from the Mental Health Screening Schedule	67,901 (sample); 296 (lifetime); age ≥15	Investigators were trained and tested together
**Sc83**	Residents of rural Changshou district of Chongqing City (2007)	Multi-stage census-based random sampling;	1. CCMD-3;<br>2. Additional tools:<br>a. The record list for diagnosis of psychosis;<br>b. PSE;<br>c. SDSS;<br>d. National Sampling Surveys Standard of Disability of 1986;	Medical doctors	12,876 (sample); 71 (lifetime); 62 (point); age ≥15	All assessors underwent unified training and their application of assessment tools had good consistency
**Sc85**	Residents of Pu'er City (urban); residents of	Multistage cluster and stratified random	1. CCMD-3;<br>2. ICD-10;<br>3. The Manual of Psychiatric Epidemiological Survey, 1985;	Investigators were divided into three groups, each comprised of three	14,424 (sample); 78 (lifetime); 63 (point);	Pre-survey training (Kappa=0·92)

	Jinggu and Jiangcheng country (rural) (2006)	sampling		psychiatrists with more than 10 years of clinical experience and one senior physician	age ≥15	
**Sc90**	Residents of urban and rural Shantou (1995)	Census-based random sampling and door-to door survey	1. CCMD-2-R;<br>2. Additional tools:<br>a. Psychological health screening scale;<br>b. PSE-9;<br>c. Schizophrenia mental history form	The investigation team were made up of five attending psychiatrists, one physician, three nurses and one staff member in charge of psychological tests. Diagnoses were confirmed by two attending physicians.	3,320 (sample);<br>31 (lifetime);<br>26 (point);<br>age ≥15	Pre-survey training (Kappa=92·1-98·8%).
**Sc91**	Residents of urban and rural Shangrao (2002)	Probability proportional to size stratified and cluster random sampling	1. ICD-10;<br>2. CCMD-3;<br>3. Additional tools:<br>a. CIDI;<br>b. SDSS;<br>c. Adult Mental Disability Rating Scales;<br>d. Children's intelligence screening test of 40 questions;<br>e. Wechsler Intelligence Scale for Children, Chinese version (C-WISE);<br>f. Crichton royal behavioural rating scale (CRBRS);<br>g. Activity of Daily Living Scale (ADL);<br>h. Hachinski Ischemic Score (HIS);<br>i. Social-demographic information registration forms;<br>j. Recording sheet of various psychiatric history;	A 12-member investigation team was divided into four subgroups; each consisted of one psychiatrist, and two senior nurses.	2,653 (sample);<br>25 (lifetime);<br>18 (point);<br>age ≥15	Pre-survey training (Kappa =0.53-0.92); review of 5% households with negative results, found no false-negative cases.

**Sc92a**	In 1991: residents of 4 streets in an urban area;<br>In 2001: residents of 6 streets in urban area and 5 townships in village area (1991, 2001)	Stratified and cluster random sampling	1. CCMD-II and CCMD-II-R<br>2. Additional tools:<br>a. Investigation questionnaire of clues to mental diseases;<br>b. Mental diseases patient related information questionnaire;<br>c. Mental health screening scale<br>d. Neurosis screening scale;<br>e. SDSS;<br>f. Wechsler Preschool and Primary Intelligence Scale;<br>g. SANS;	1. Phase 1: Trained Investigators<br>2. Phase 2: Psychiatrists	280,878 (sample);<br>1246 (lifetime);<br>age ≥15	Investigators underwent uniform pre-survey field training (Kappa=0·7-1·0)
**Sc93**	Residents of 8 towns in Shaoxing country (2003)	Census-based random sampling	1. CCMD-3	1. Phase 1: Trained Investigators<br>2. Phase 2: Diagnostic confirmation by two psychiatrists	267,192 (sample);<br>933 (lifetime);<br>age ≥15	Uniform pre-survey field training
**Sc98**	Residents of Leshan City (2000)	Multistage and cluster random sampling	1. CCMD-2-R;<br>2. Additional tools:<br>a. Manual of Psychiatric Epidemiological Survey, 1985;<br>b. Supplement to the Manual of Psychiatric Epidemiological Survey, 1992;<br>c. Mental health screening scale;<br>d. Neurosis screening scale;<br>e. Children's intelligence screening test of 40 questions;<br>f. PSE;<br>g. SDSS;<br>h. WISC	Psychiatrists, using household survey and scores from the Mental Health Screening Schedule	3,519 (sample);<br>29 (lifetime);<br>28 (point);<br>age ≥15	Strict and uniform pre-survey training for 4 weeks (Kappa=0·68-0·92). Review of 10% of the sample for negative cases, found no false-negatives.
**Sc100**	Residents of Weihai City and rural areas in 3 Counties (2006)	Stratified and cluster random sampling	1. CCMD-3;<br>2. Additional tools:<br>a. Mental health screening scale;<br>b. Neurosis screening scale;<br>c. PSE;<br>d. PSE-54 for neurosis history;<br>e. SDSS;	Not specified in methods, but results suggest a two-stage case identification by trained investigators (Stage 1) and then	50,174 (sample);<br>258 (lifetime);<br>213 (point);<br>age ≥15	The review found no false-negative and no false-positive cases.

			f. Mini-Mental State Examination (MMSE);<br>g. Recording sheet of various psychiatric history	psychiatrists (Stage 2).		
**Sc101**	Residents of 16 administrative sub-districts in Weifang (2004)	Stratified and cluster random sampling	1. DSM-IV;<br>2. Additional tools:<br>a. Structured Clinical Interview for DSM-IV-TR<br>b. SCID-I/P;<br>c. General health questionnaire (GHQ);	A team of 7 psychiatrists and 6 nurses (or senior nurses) were divided into two groups. Schizophrenia cases reported in Group A were crossed checked by investigators in Group B, and vice versa, to prevent false positive cases.	4,763 (sample);<br>49 (lifetime);<br>46 (point);<br>age ≥18	Investigators underwent unified pre-survey training for 4 weeks, which included conformity assessment of site examination (Kappa= 0·89-0·98); review to confirm the diagnosis found no false-negative cases.
**Sc103**	Residents of urban and rural Wenzhou (2003)	Stratified and cluster random sampling	1. ICD-10;<br>2. CCMD-3;<br>3. Additional tools:<br>a. Psychological health screening scale;<br>b. Present State Examination (PSE-140);<br>c. Mental health questionnaire;<br>d. Household socio-demographic registration form;	The 14-member investigation team was divided into 4 subgroups; each included at least 1 medical doctor and 2 nurses. Those with a score of 2 point or above on the "Psychological health screening scale" were then asked to fill in a PSE.	18,173 (sample);<br>134 (lifetime);<br>109 (point);<br>age ≥15	Consistency test of the screening and diagnostic tools, Kappa index=0·53-0·92 (P>0·05)
**Sc106**	Residents of four	Stratified and cluster	1. DSM-IV;<br>2. Additional tools:	A core investigation was made up of	5,375 (sample);<br>20 (lifetime);	All investigation tools were

	representa-tive areas of Tibet (Lhasa, Shigatse Prefecture, Naqu District and Linzhi Area) (2003)	random sampling	a. The Manual of Psychiatric Epidemiological Survey, 1985;<br>b. Mental health screening scale;<br>c. SCID-I;<br>d. Recording sheet of mental disorders history;<br>e. Social-demographic information registration forms;	eight members from the Institute of Psychological Health Research in Huaxi Hospital of Sichuan University (including two professors, three chief physicals, and three research students). The team in Tibet also included seven non-medical staff, six of who were Tibetans. Each investigation subgroup was made up of one psychiatrist and one local Tibetan investigator.	18 (point);<br>age ≥15	translated into standard Tibetan; investigators underwent pre-survey training (Kappa=0·68-0·82). Review of 10% negative subjects identified false-negative rate of 0·02.
**Sc109**	Residents of Xinshi District, Urumqi (1995)	Census-based random sampling	1. CCMD-II-R;<br>2. Mental health screening scale;<br>3. Handbook of prevention and rehabilitation of mental diseases;<br>4. Disability rating standard from the handbook of national sampling survey of disability;	Phase 1 - Screening: by 300 medical and non-medical staff members from seven sub-district offices;<br>Phase 2 – Diagnosis by psychiatrists.	190,683 (sample);<br>667 (lifetime);<br>617 (point);<br>age ≥15	Two pre-survey training courses of staff (Kappa= 0·75) and psychiatrists (Kappa =0·74).
**Sc110**	Residents of rural areas of Xinjin County (1994)	Census-based random sampling	1. ICD-10<br>2. CCMD-2-R	1. Phase 1: face-to-face interviews of all heads of households and village doctors by trained investigators<br>2. Phase 2: all	123,572 (sample), 510 (lifetime cases), 376 (point cases), age ≥15	Pre-survey training (ICC=91·0%; Kappa =0·7-1·0)

				suspected cases of schizophrenia were interviewed by psychiatrists		
**Sc111**	Residents of four districts in Xin Xiang city (Xinhua, Hongqi, Beizhan, Jiaoqu) (2000)	Stratified and cluster random sampling	1. CCMD;<br>2. Manual of psychopathy epidemiology survey;<br>3. Mental health screening scale;<br>4. Neurosis screening schedule;<br>5. Children's intelligence screening test;<br>6. SDSS;	Not specified in methods, but results suggest a two-stage case identification by trained investigators (Stage 1) and then psychiatrists (Stage 2).	606,762 (sample);<br>2,457 (lifetime);<br>373 (point);<br>age ≥15	Review of 10% negative subjects to assess false-negative rate.
**Sc112**	Residents of 9 Hani villages in Yuan Yang (2009)	Cluster random sampling	1. ICD-10	Investigators were psychiatrists, who conducted household interviews with suspected cases of schizophrenia identified by village doctors, and other health personnel.	12,581 (sample);<br>39 (lifetime);<br>33 (point);<br>age ≥15	Not specified
**Sc118**	Residents of Beijing, 2003	Stratified random sampling	1. ICD-10<br>2. Composite International Diagnostic Interview, Chinese Version 1·0 (CIDI 1·0)	A large team of 102 psychiatrists	5,926 (sample);<br>31 (lifetime);<br>age ≥15	Training in epidemiological fieldwork and a 10-day workshop by a WHO-qualified trainer (Kappa=0·795)

Table s4. Distribution of studies by province/municipalities.

Provinces/ municipalities	Number of publications containing prevalence data
Anhui	1
Beijing	4
Guangdong	5
Guangxi	3
Guizhou	1
Hainan	2
Heilongjiang	2
Henan	1
Hubei	2
Hunan	1
Jiangsu	4
Jiangxi	3
Jilin	1
Liaoning	1
Shandong	4
Shanghai	1
Sichuan	4
Tibet	1
Yunnan	3
Xinjiang	1
Zhejiang	3
TOTAL	48*

*Note: 1 of the prevalence studies was conducted in 7 provinces/municipalities.

Table s5. The table presents a targeted sub-analysis of the data to avoid potential differences in study design and case ascertainment between urban and rural areas. A specific comparison of prevalence of schizophrenia in urban and rural setting is presented for 10 studies that used a sample of comparable size from both urban and rural area within the same setting, and used the same study design and methods of case ascertainment.

Study	Urban cases, sample size and point prevalence			Rural cases, sample size and point prevalence		
Sc 13	15	3794	0.0040	16	2168	0.0074
Sc 14	53	8104	0.0065	42	9677	0.0043
Sc 23	26	5355	0.0049	22	6585	0.0033
Sc 27	131	19276	0.0068	87	16109	0.0054
Sc 33	78	11632	0.0067	59	21700	0.0027
Sc 47	10	1310	0.0076	10	1599	0.0063
Sc 52	61	7028	0.0087	14	2200	0.0064
Sc 85	34	7546	0.0045	29	6878	0.0042
Sc 90	12	1542	0.0078	14	1778	0.0079
Sc 103	60	9338	0.0064	49	8835	0.0055
	480	74925	0.0064	342	77529	0.0044

Figure s1a-c. Meta-analysis of the retained studies to explore the effects of urban area residence, year of study and method of case finding, to explore the effects of study heterogeneity. All results are based on prevalence estimates per 1000 population (to make the graphs more presentable). In all analyses, a random effects model was used because of high heterogeneity. Results of heterogeneity are reported in the graph (I^2 and p-value). When I^2 is higher than 50% and p-value is less than 0.05, there is an evidence of heterogeneity.

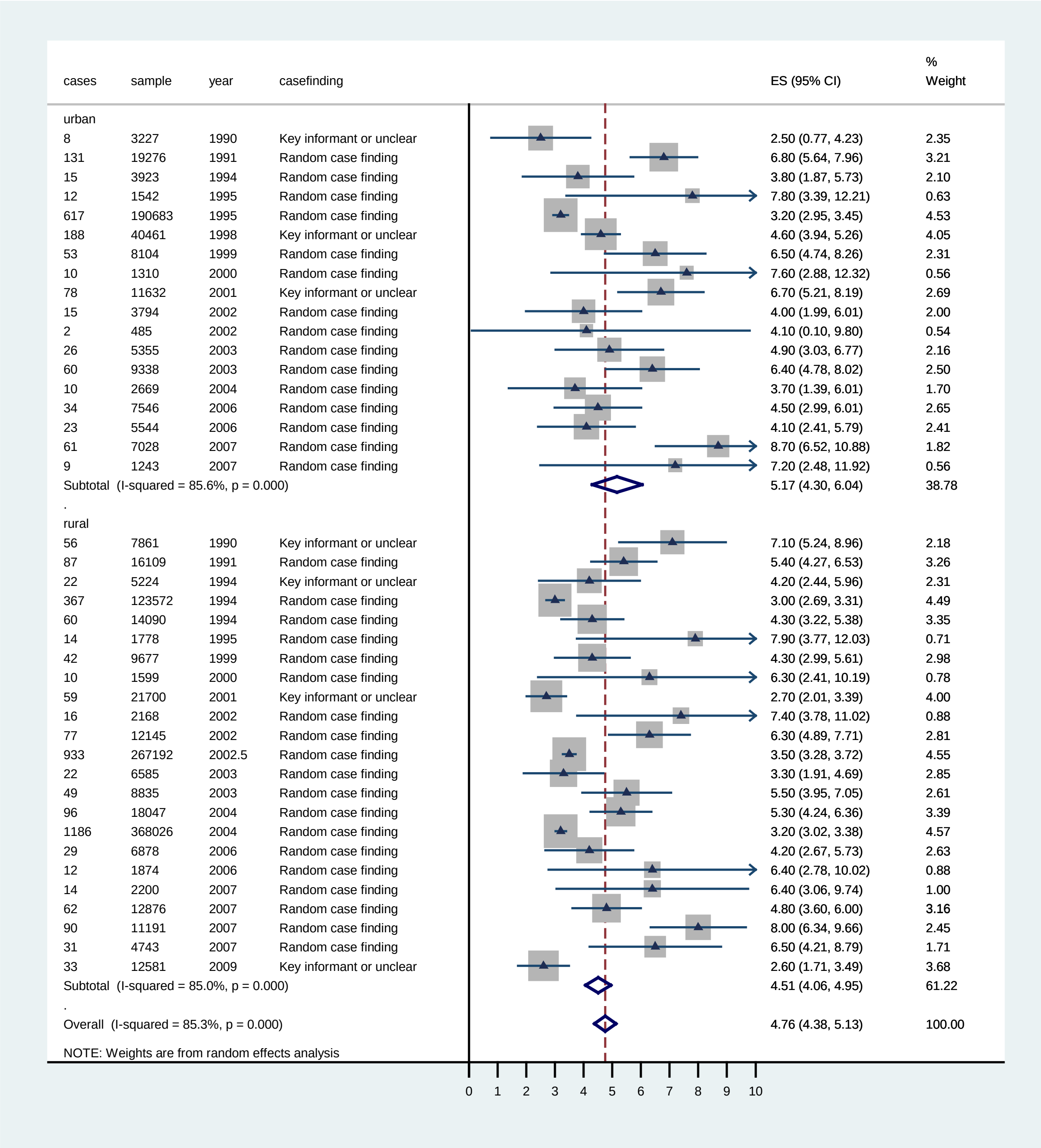

Figure s1a

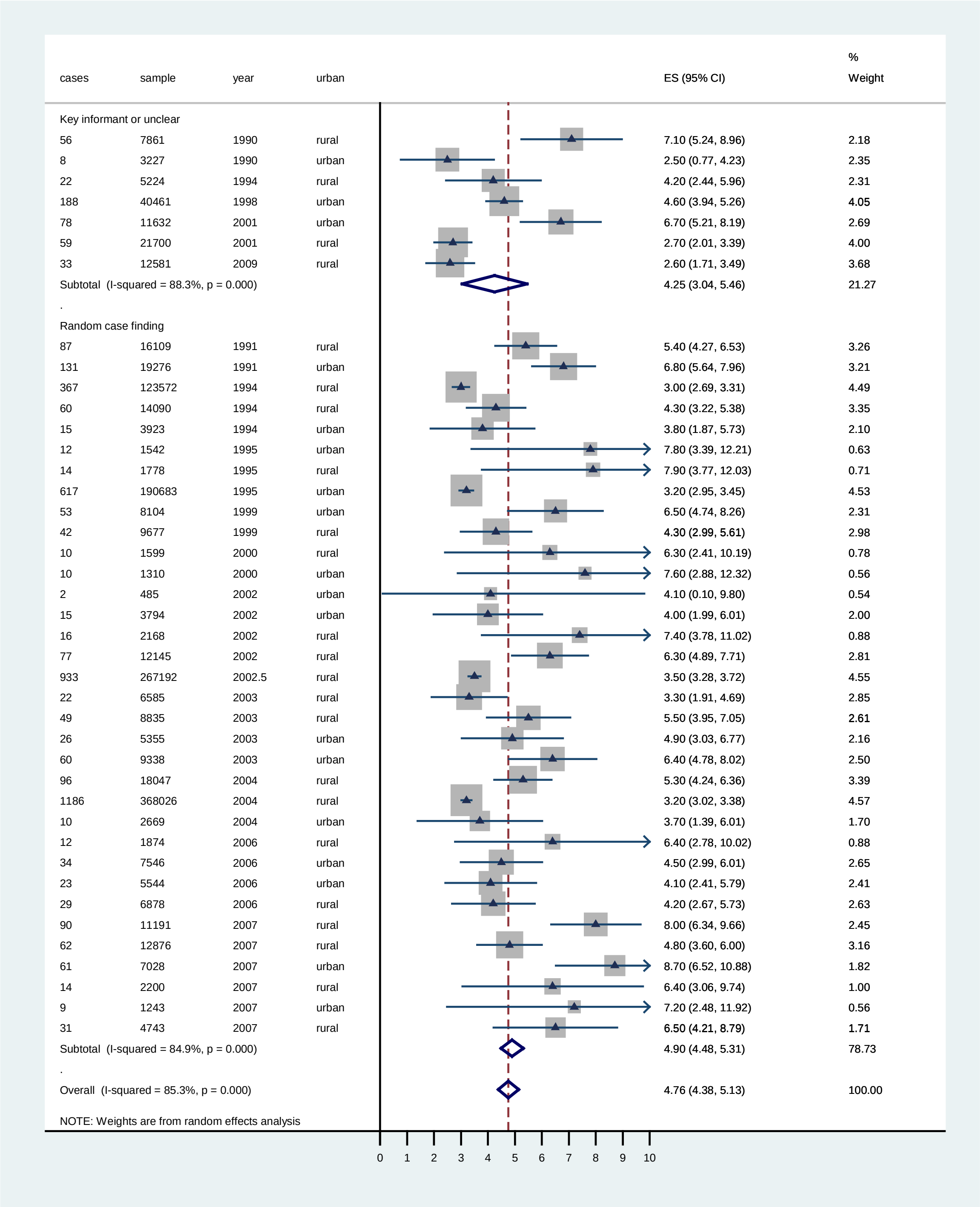
cases sample year urban ES (95% CI) % Weight
Key informant or unclear
56 7861 1990 rural 7.10 (5.24, 8.96) 2.18
8 3227 1990 urban 2.50 (0.77, 4.23) 2.35
22 5224 1994 rural 4.20 (2.44, 5.96) 2.31
188 40461 1998 urban 4.60 (3.94, 5.26) 4.05
78 11632 2001 urban 6.70 (5.21, 8.19) 2.69
59 21700 2001 rural 2.70 (2.01, 3.39) 4.00
33 12581 2009 rural 2.60 (1.71, 3.49) 3.68
Subtotal (I-squared = 88.3%, p = 0.000) 4.25 (3.04, 5.46) 21.27
.
Random case finding
87 16109 1991 rural 5.40 (4.27, 6.53) 3.26
131 19276 1991 urban 6.80 (5.64, 7.96) 3.21
367 123572 1994 rural 3.00 (2.69, 3.31) 4.49
60 14090 1994 rural 4.30 (3.22, 5.38) 3.35
15 3923 1994 urban 3.80 (1.87, 5.73) 2.10
12 1542 1995 urban 7.80 (3.39, 12.21) 0.63
14 1778 1995 rural 7.90 (3.77, 12.03) 0.71
617 190683 1995 urban 3.20 (2.95, 3.45) 4.53
53 8104 1999 urban 6.50 (4.74, 8.26) 2.31
42 9677 1999 rural 4.30 (2.99, 5.61) 2.98
10 1599 2000 rural 6.30 (2.41, 10.19) 0.78
10 1310 2000 urban 7.60 (2.88, 12.32) 0.56
2 485 2002 urban 4.10 (0.10, 9.80) 0.54
15 3794 2002 urban 4.00 (1.99, 6.01) 2.00
16 2168 2002 rural 7.40 (3.78, 11.02) 0.88
77 12145 2002 rural 6.30 (4.89, 7.71) 2.81
933 267192 2002.5 rural 3.50 (3.28, 3.72) 4.55
22 6585 2003 rural 3.30 (1.91, 4.69) 2.85
49 8835 2003 rural 5.50 (3.95, 7.05) 2.61
26 5355 2003 urban 4.90 (3.03, 6.77) 2.16
60 9338 2003 urban 6.40 (4.78, 8.02) 2.50
96 18047 2004 rural 5.30 (4.24, 6.36) 3.39
1186 368026 2004 rural 3.20 (3.02, 3.38) 4.57
10 2669 2004 urban 3.70 (1.39, 6.01) 1.70
12 1874 2006 rural 6.40 (2.78, 10.02) 0.88
34 7546 2006 urban 4.50 (2.99, 6.01) 2.65
23 5544 2006 urban 4.10 (2.41, 5.79) 2.41
29 6878 2006 rural 4.20 (2.67, 5.73) 2.63
90 11191 2007 rural 8.00 (6.34, 9.66) 2.45
62 12876 2007 rural 4.80 (3.60, 6.00) 3.16
61 7028 2007 urban 8.70 (6.52, 10.88) 1.82
14 2200 2007 rural 6.40 (3.06, 9.74) 1.00
9 1243 2007 urban 7.20 (2.48, 11.92) 0.56
31 4743 2007 rural 6.50 (4.21, 8.79) 1.71
Subtotal (I-squared = 84.9%, p = 0.000) 4.90 (4.48, 5.31) 78.73
.
Overall (I-squared = 85.3%, p = 0.000) 4.76 (4.38, 5.13) 100.00
NOTE: Weights are from random effects analysis
0 1 2 3 4 5 6 7 8 9 10

Figure s1b

cases	sample	year	urban	casefinding	ES (95% CI)	% Weight
1990-2000						
8	3227	1990	urban	Key informant or unclear	2.50 (0.77, 4.23)	2.35
56	7861	1990	rural	Key informant or unclear	7.10 (5.24, 8.96)	2.18
131	19276	1991	urban	Random case finding	6.80 (5.64, 7.96)	3.21
87	16109	1991	rural	Random case finding	5.40 (4.27, 6.53)	3.26
15	3923	1994	urban	Random case finding	3.80 (1.87, 5.73)	2.10
367	123572	1994	rural	Random case finding	3.00 (2.69, 3.31)	4.49
60	14090	1994	rural	Random case finding	4.30 (3.22, 5.38)	3.35
22	5224	1994	rural	Key informant or unclear	4.20 (2.44, 5.96)	2.31
12	1542	1995	urban	Random case finding	7.80 (3.39, 12.21)	0.63
617	190683	1995	urban	Random case finding	3.20 (2.95, 3.45)	4.53
14	1778	1995	rural	Random case finding	7.90 (3.77, 12.03)	0.71
188	40461	1998	urban	Key informant or unclear	4.60 (3.94, 5.26)	4.05
53	8104	1999	urban	Random case finding	6.50 (4.74, 8.26)	2.31
42	9677	1999	rural	Random case finding	4.30 (2.99, 5.61)	2.98
10	1310	2000	urban	Random case finding	7.60 (2.88, 12.32)	0.56
10	1599	2000	rural	Random case finding	6.30 (2.41, 10.19)	0.78
Subtotal (I-squared = 86.6%, p = 0.000)					4.77 (4.09, 5.44)	39.81
.						
2001-2009						
78	11632	2001	urban	Key informant or unclear	6.70 (5.21, 8.19)	2.69
59	21700	2001	rural	Key informant or unclear	2.70 (2.01, 3.39)	4.00
15	3794	2002	urban	Random case finding	4.00 (1.99, 6.01)	2.00
2	485	2002	urban	Random case finding	4.10 (0.10, 9.80)	0.54
77	12145	2002	rural	Random case finding	6.30 (4.89, 7.71)	2.81
16	2168	2002	rural	Random case finding	7.40 (3.78, 11.02)	0.88
933	267192	2002.5	rural	Random case finding	3.50 (3.28, 3.72)	4.55
60	9338	2003	urban	Random case finding	6.40 (4.78, 8.02)	2.50
26	5355	2003	urban	Random case finding	4.90 (3.03, 6.77)	2.16
49	8835	2003	rural	Random case finding	5.50 (3.95, 7.05)	2.61
22	6585	2003	rural	Random case finding	3.30 (1.91, 4.69)	2.85
10	2669	2004	urban	Random case finding	3.70 (1.39, 6.01)	1.70
96	18047	2004	rural	Random case finding	5.30 (4.24, 6.36)	3.39
1186	368026	2004	rural	Random case finding	3.20 (3.02, 3.38)	4.57
23	5544	2006	urban	Random case finding	4.10 (2.41, 5.79)	2.41
34	7546	2006	urban	Random case finding	4.50 (2.99, 6.01)	2.65
29	6878	2006	rural	Random case finding	4.20 (2.67, 5.73)	2.63
12	1874	2006	rural	Random case finding	6.40 (2.78, 10.02)	0.88
9	1243	2007	urban	Random case finding	7.20 (2.48, 11.92)	0.56
61	7028	2007	urban	Random case finding	8.70 (6.52, 10.88)	1.82
14	2200	2007	rural	Random case finding	6.40 (3.06, 9.74)	1.00
31	4743	2007	rural	Random case finding	6.50 (4.21, 8.79)	1.71
62	12876	2007	rural	Random case finding	4.80 (3.60, 6.00)	3.16
90	11191	2007	rural	Random case finding	8.00 (6.34, 9.66)	2.45
33	12581	2009	rural	Key informant or unclear	2.60 (1.71, 3.49)	3.68
Subtotal (I-squared = 85.1%, p = 0.000)					4.83 (4.32, 5.34)	60.19
.						
Overall (I-squared = 85.3%, p = 0.000)					4.76 (4.38, 5.13)	100.00

NOTE: Weights are from random effects analysis

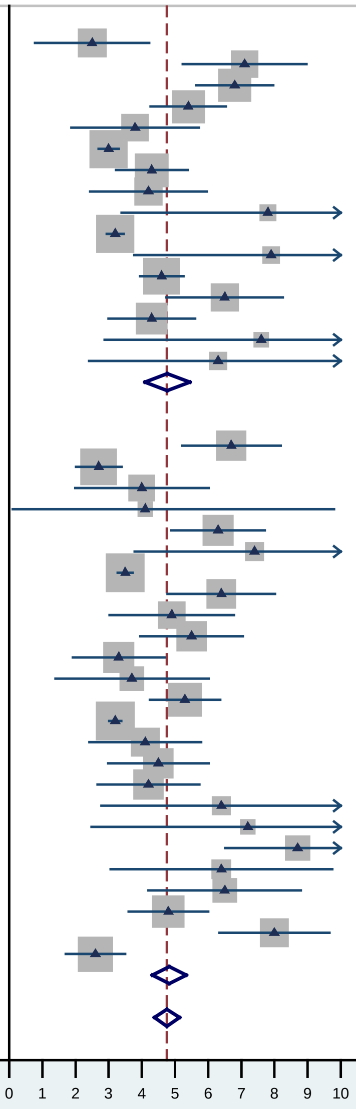

Figure s1c

eMethods. Statistical analyses of the data.

1. Bayesian analysis to estimate the prevalence in urban and rural regions at different time points

The results here are based on a Bayesian analysis. Based on the data available, we have used a binomial logistic regression model. As we have used the same approach for all four datasets, we describe it for the lifetime urban studies.

Let y_i denote the number of cases of schizophrenia for study i = 1, 2, . . . , 28 (because 28 of 42 studies contained information on urban areas and lifetime prevalence), and N_i denote the total population size. Then the model states that

$$y_i \sim \text{Binomial}(N_i, p_i).$$

The unknown parameters p_i are the unknown probabilities of an individual selected at random having schizophrenia for each study. These would be modeled on the number of years since the earliest study using the logistic link. This means

$$\text{logit}(p_i) = \log\left(\frac{p_i}{1-p_i}\right) = \beta_0 + \beta_1 \times \text{time}_i,$$

or equivalently

$$p_i = \frac{\exp\{\beta_0 + \beta_1 \times \text{time}_i\}}{1 + \exp\{\beta_0 + \beta_1 \times \text{time}_i\}}.$$

In this equation, time_i is 0 for any of the studies in 1990 (in this case) and represents the number of years since 1990 and β_0 and β_1 represent unknown parameters to make inference on. The Bayesian approach allows prior knowledge (if any) to be incorporated. As we didn't have any prior knowledge, we express this lack of knowledge using independent normal distributions on β_0 and β_1 both having a huge variance of 10^6:

$$\beta_0 \sim \text{Normal}(0, 10^6),$$
$$\beta_1 \sim \text{Normal}(0, 10^6).$$

Now using Bayes theorem, it was possible to find the joint distribution of β_0 and β_1 given the data that was observed. This is called the posterior distribution and is denoted as

$$\Pr(\beta_0, \beta_1 | y_1, y_2, \ldots, y_{28})$$

or as $\Pr(\beta_0, \beta_1|y)$ for short. Of particular interest were the marginal posterior distributions $\Pr(\beta_0|y)$ and $\Pr(\beta_1|y)$ which can be obtained from $\Pr(\beta_0, \beta_1|y)$ by integration.

Given the marginal posterior distribution, appropriate summaries could be obtained. These could include point estimates such as the posterior mean, median or mode as well as uncertainty measures including the standard deviation and the 95% credible interval defined for β_0 as the interval (l, u) such that

$$\Pr(l < \beta_0 < u|\mathbf{y}) = 0.95.$$

To obtain the quantities above, it was necessary to use Markov chain Monte Carlo sampling, because the relevant marginal posterior distributions were not available in closed form. Given

some initial values, this sampling scheme runs through 2 stages: the "burn in", which must be discarded, and the "post burn in" which can be retained as approximate samples from the marginal posterior distributions of interest. The relevant quantities can then be calculated on these samples.

We have used a Markov chain Monte Carlo sampler called a Gibbs sampler. Based on 3 independent chains, we found no evidence of lack of convergence after 1,000 iterations, so this part of the sample was discarded. The results in **Table S1** were based on pooling the samples from each chain after discarding 3,000 burn in samples (1,000 per chain) and leaving a total sample of 30,000 samples. We used the sample posterior median as the estimate.

It is often of interest to compare two or more competing models. There are a number of approaches to do this in a Bayesian framework. One popular approach is based on the deviance information criterion (DIC) introduced in Spiegelhalter et al. (2002). This popularity is in part due to the fact that it can be estimated from a sample from the posterior distribution. In common with other model selection criteria, it consists of a measure of fit to the data (the deviance) and a penalty on model complexity to guard against over-fitting. In comparing two or more models, the 'best' model is the one with the smallest value of the DIC. The results in **Table S2** are the difference in DIC between the model with time as a covariate and the intercept only model.

The results suggested that for studies in the 'lifetime' category, the log odds ratio for both rural and urban studies was positive. The credible intervals did not contain 0 and the deviance information criterion was lower for the model including time, implying that there was indeed an increase in probability of schizophrenia as time increases. For the studies in the 'point' category, the probability of schizophrenia in urban areas also appeared to increase over the years, but for rural areas, the credible interval included 0 and the DIC for the intercept only model was smaller, suggesting that the model stated that the probability of schizophrenia is constant in rural areas.

eMethods Table 1. Results from Bayesian analysis of schizophrenia studies. The estimate is based on the posterior median. The covariate "Year (of study)" is included as number of years since the earliest study.

Outcome	Residency	Covariate	Estimate	95% Credible Interval
Lifetime prevalence	Urban	Intercept	-5·555	(-5·608, -5·486)
		Year	0·038	(0·031, 0·045)
	Rural	Intercept	-5·597	(-5·683, -5·513)
		Year	0·015	(0·009, 0·022)
Point prevalence	Urban	Intercept	-5·738	(-5·846, -5·682)
		Year	0·038	(0·025, 0·051)
	Rural	Intercept	-5·582	(-5·696, -5·479)
		Year	-0·003	(-0·010, 0·005)

eMethods Table 2. The deviance information criterion (DIC) difference relative to the intercept only model (i.e. DIC for model with year of study as a covariate - DIC for intercept only model).

Outcome	Residency	DIC difference
Lifetime prevalence	Urban	105·50
	Rural	19·87
Point prevalence	Urban	28·81
	Rural	-1·54

Based on the samples, it was possible to estimate the probabilities of having schizophrenia in 1990, 2000 and 2010, together with a 95% credible intervals, as presented in **Table 2** in the main text. Credible intervals did not take into account the likely effect of sampling uncertainty, which - if taken into account - would slightly expand the credible interval.

2. Additional sensitivity analysis to investigate the potential effects of age and sex distribution of the study sample on the prevalence of schizophrenia

Our primary interest in this study was to explore the effects of the year of study and urban/rural residency on the prevalence of schizophrenia. We based our analysis on 42 large studies, all of which provided the information on predictor variables - year of study and urban/rural setting. Our primary analysis resulted in robust and internally consistent estimates, with narrow confidence intervals, which was expected given a very large overall sample size (2·28 million examinees).

Following the completion of our primary analysis (above), we run an additional sensitivity analysis to explore a potential bias that could have arisen from possible differences in age and sex distribution of the examinees between the samples of different studies. We did not include mean age or male-to-female ratio in the primary analysis for three reasons:

i. We did not have complete information on age and/or sex distribution of the sample from a number of studies;
ii. Reports on prevalence specifically by gender were available only from a handful of studies; and
iii. Adding of additional covariates to the Bayesian analysis described above leads to large increase in the complexity of computations and demand for computer time and capacity.

We did not expect the effect of internal age-sex structure of the samples to be a major confounding factor in our study because:

i. The samples in all studies were large (or very large) and broadly representative of the underlying population; male-to-female ratio from the samples was therefore similar to that expected in the population, which meant that the observed prevalence could not be dramatically affected by male-to-female ratio of the sample, even if there were significant differences in prevalence between two sexes;
ii. Lifetime morbid risk for schizophrenia does not have dramatic peaks at particular ages, so it was unlikely that the mean age of the sample would be a striking predictor of the prevalence, necessitating adjustments in the observed reports before the final analysis.

We used the same analysis described above, based on the deviance information criterion (DIC) (Spiegelhalter et al., 2002), to explore the role of the mean age of the sample and the sex on the prevalence of schizophrenia in a limited sub-sample where this information was available. The results are shown in **Table S3**:

Table s3. The deviance information criterion (DIC) for full model, including the information on age of examinees (Age), year of study (YoS), urban/rural residency (Res) and gender (Sex).

	Deviance	Penalty	DIC
DIC - effect of mean age on lifetime prevalence			
Full model	293·61	2·97	296·58
No [Age]	300·14	2·01	302·15
DIC - effect of mean age on point prevalence			
Full model	277·52	3·01	280·52
No [Age]	278·16	2·01	280·17
DIC - effect of gender on lifetime prevalence			
Full Model	601·4	8·0	609·4
No [Sex]	701·7	3·9	705·7
No [Res]	672·1	3·9	676·1
No [YoS]	772·0	4·0	776·1
DIC - effect of gender on point prevalence			

Full Model	280·7	8·0	288·7
No [Sex]	370·6	4·0	374·5
No [Res]	391·7	3·9	395·6
No [YoS]	356·5	4·1	360·6

The analysis shows that DIC does not increase when mean age is dropped as a predictor, meaning that mean age of the sample has no effect on the reported prevalence. However, the analysis in a sub-sample of studies where the prevalence was reported differentially by sex indicates that there are differences in prevalence by sex, with males having higher rates. However, this does not affect our population-based estimates for China, because the male-to-female ratio in our samples was comparable to the male-to-female ratio in Chinese population aged 15 years or more, to which we applied the estimates of the prevalence, meaning that no further adjustments were necessary.

eReference

Spiegelhalter DJ, Best NG, Carlin BP, van der Linde A. Bayesian measures of model complexity and fit (with discussion). *J R Stat Soc Series B Stat Methodol.* 2002;64:583-639.
